# Endometriosis as a Systemic and Complex Disease: Toward Phenotype-Based Classification and Personalized Therapy

**DOI:** 10.3390/ijms27020908

**Published:** 2026-01-16

**Authors:** Daniel Simancas-Racines, Emilia Jiménez-Flores, Martha Montalvan, Raquel Horowitz, Valeria Araujo, Claudia Reytor-González

**Affiliations:** 1 Facultad de Ciencias de la Salud y Bienestar Humano, Universidad Tecnológica Indoamérica, Ambato 180150, Ecuador; emily-j01@hotmail.com; 2 Facultad de Salud y Bienestar, Pontificia Universidad Católica del Ecuador, Quito 170143, Ecuador; 3 Centro de Investigación en Salud Pública y Epidemiología Clínica (CISPEC), Facultad de Ciencias de la Salud Eugenio Espejo, Universidad UTE, Quito 170527, Ecuador; 4 Escuela de Medicina, Universidad Espíritu Santo, Samborondón 0901952, Ecuador; 5 Department of Medicine, Geriatrics Division, Montefiore Medical Center, Bronx, NY 10467, USA; rahorowitz@montefiore.org; 6 Escuela de Medicina, Pontificia Universidad Católica del Ecuador, Santo Domingo 230203, Ecuador

**Keywords:** endometriosis, immune crosstalk, epigenetic regulation, chronic inflammation, molecular biomarkers, precision medicine

## Abstract

Endometriosis is traditionally conceptualized as a pelvic lesion–centered disease; however, mounting evidence indicates it is a chronic, systemic, and multifactorial inflammatory disorder. This review examines the molecular dialog between ectopic endometrial tissue, the immune system, and peripheral organs, highlighting mechanisms that underlie disease chronicity, symptom variability, and therapeutic resistance. Ectopic endometrium exhibits distinct transcriptomic and epigenetic signatures, disrupted hormonal signaling, and a pro-inflammatory microenvironment characterized by inflammatory mediators, prostaglandins, and matrix metalloproteinases. Immune-endometrial crosstalk fosters immune evasion through altered cytokine profiles, extracellular vesicles, immune checkpoint molecules, and immunomodulatory microRNAs, enabling lesion persistence. Beyond the pelvis, systemic low-grade inflammation, circulating cytokines, and microRNAs reflect a molecular spillover that contributes to chronic pain, fatigue, hypothalamic–pituitary–adrenal axis dysregulation, and emerging gut–endometrium interactions. Furthermore, circulating biomarkers—including microRNAs, lncRNAs, extracellular vesicles, and proteomic signatures—offer potential for early diagnosis, patient stratification, and monitoring of therapeutic responses. Conventional hormonal therapies demonstrate limited efficacy, whereas novel molecular targets and delivery systems, including angiogenesis inhibitors, immune modulators, epigenetic regulators, and nanotherapeutics, show promise for precision intervention. A systems medicine framework, integrating multi-omics analyses and network-based approaches, supports reconceptualizing endometriosis as a systemic inflammatory condition with gynecologic manifestations. This perspective emphasizes the need for interdisciplinary collaboration to advance diagnostics, therapeutics, and individualized patient care, ultimately moving beyond a lesion-centered paradigm toward a molecularly informed, holistic understanding of endometriosis.

## 1. Introduction

Endometriosis is a chronic, estrogen-dependent inflammatory disorder defined by the presence of endometrial-like glands and stroma outside the uterine cavity, provoking sustained inflammation and commonly associated with pain and infertility. Globally, it affects roughly 10–15% of women of reproductive age, with an estimated 9 million cases in the United States, and prevalence reaching up to 70% among individuals with chronic pelvic pain [1]. U.S. hospital data indicate that 11.2% of women aged 18–45 admitted for genitourinary concerns and 10.3% undergoing gynecologic surgery receive an endometriosis diagnosis [2]. Beyond clinical morbidity, endometriosis imposes significant economic burdens, with average annual per-patient costs around €10,000 in Europe and higher expenditures in the United States of America [3].

Clinically, the condition is primarily associated with pelvic pain, including dysmenorrhea, dyspareunia, and discomfort without menstruation, reported by nearly 90% of patients, alongside infertility affecting up to 50% of those diagnosed [2,4]. Lesions most frequently involve the ovaries, uterosacral ligaments, and peritoneum but may extend to extrapelvic sites such as the urinary tract, intestines, pleura, pericardium, and central nervous system [5]. Diagnosis is often delayed, averaging 5–12 years from symptom onset, with patients consulting multiple providers before confirmation, which remains largely dependent on laparoscopic visualization, despite increased use of transvaginal ultrasound and pelvic magnetic resonance imaging (MRI) [6]. Risk factors include early menarche, short menstrual cycles, heavy menstrual bleeding, and nulliparity, whereas protective factors encompass parity, extended breastfeeding, hormonal contraceptive use, tubal ligation, regular physical activity, and dietary omega-3 intake [1].

**Figure 1:**
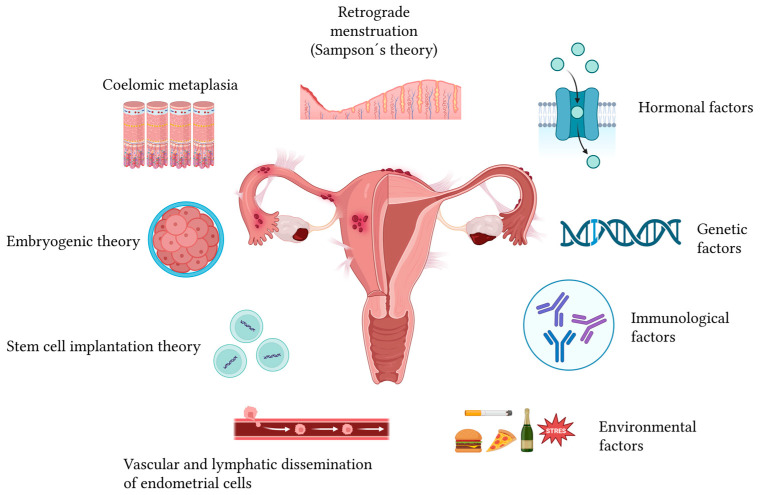
Possible mechanisms underlying the pathophysiology of endometriosis and the primary factors contributing to its development. Since the pathophysiology of endometriosis is not yet fully understood due to its complexity and multifactorial nature, several hypotheses have been proposed to explain its development, including the theories of retrograde menstruation, coelomic metaplasia, embryogenic origin, stem cell implantation, and vascular or lymphatic dissemination of endometrial cells. Factors involved in its progression include hormonal, genetic, immunological, and environmental influences—such as poor nutrition (including processed foods), tobacco use, excessive alcohol consumption, and chronic stress—which act as aggravating elements that may intensify inflammation and promote disease advancement [17,18,19]. Created in BioRender. Reytor, C. (2026) https://BioRender.com/0z5en66 (accessed on 12 January 2026).

The pathogenesis of endometriosis is multifactorial (Figure 1). While Sampson’s retrograde menstruation theory is widely referenced, it alone does not account for the disease, as retrograde flow occurs in many women without pathology [5]. Additional mechanisms—coelomic metaplasia, Müllerian remnants, vascular or lymphatic dissemination, stem cell contribution, oxidative stress, chronic inflammation, and genetic and epigenetic factors—further explain the systemic and heterogeneous nature of the disorder [7].

Traditional lesion-centered approaches face limitations, particularly in surgically accessing deep lesions near the bowel or bladder, which raises the risk of inadvertent injury [8]. Two-dimensional imaging may inadequately capture vascular supply or precise relationships with adjacent organs [8,9], and ultrasound or computed tomography can be insufficient for complex lesions near gas-filled structures [8,10,11,12]. These constraints underscore the utility of advanced imaging modalities, including multiplanar MRI and 3D reconstruction, which improve lesion visualization, surgical mapping, and planning [8,9].

At the molecular level, endometriosis is marked by dysregulated cellular signaling, immune dysfunction, and hormone resistance, supporting ectopic tissue survival. Critical pathways include phosphoinositide 3-kinase (PI3K)/protein kinase B (Akt) and Wingless-related integration site (Wnt)/β-catenin, which enhance proliferation and survival, alongside nuclear factor kappa-light-chain-enhancer of activated B cells (NF-κB), a central mediator of chronic inflammation [13,14]. Epigenetic changes, such as cytosine-phosphate-guanine (CpG) methylation, and genetic predisposition further reinforce disease establishment and persistence [15]. Key molecular processes involve aberrant cell signaling, immune evasion, hormonal imbalance, epigenetic regulation, oxidative stress, inflammation, tissue invasion, and fibrosis [7,13,16].

This review aims to examine the systemic nature of endometriosis, emphasizing molecular crosstalk and its implications for chronicity, clinical manifestations, and potential therapeutic strategies.

## 2. The Molecular Identity of Ectopic Endometrial Tissue

The molecular landscape of endometriotic lesions reveals a unique identity that distinguishes ectopic endometrial tissue from its eutopic counterpart. Although these lesions share histological similarities with the normal endometrium, they display specific transcriptomic, epigenetic, and hormonal alterations that underlie their survival, pro-inflammatory profile, and reduced responsiveness to conventional therapies [20]. Elucidating these molecular distinctions is essential for identifying novel therapeutic targets and improving disease management.

### 2.1. Transcriptomic and Epigenetic Profiles of Ectopic vs. Eutopic Endometrial Cells

Epigenetics refers to inheritable alterations in gene regulation that occur without changes in the DNA sequence, functioning as key modulators of cellular activity. These mechanisms include DNA methylation, histone modifications such as acetylation, phosphorylation, and methylation, as well as non-coding RNAs. Together, they remodel chromatin architecture and influence gene activation or repression. In parallel, transcriptomics provides a global view of RNA transcripts in cells or tissues, uncovering divergences between healthy and diseased states and offering insights into the molecular mechanisms of endometriosis [21].

Comparative transcriptomic studies demonstrate marked differences between ectopic and eutopic endometrium. Ectopic lesions upregulate genes promoting proliferation, angiogenesis, and inflammation, including vascular endothelial growth factor (VEGF) and interleukin-8 (IL-8), supporting lesion growth and invasiveness [22]. By contrast, eutopic endometrium retains gene programs linked to cyclic remodeling and receptivity. Co-expression analyses highlight this divergence: eutopic cells enrich adhesion and implantation pathways, while ectopic tissue displays enrichment in migration, epithelial maturation, and gap junction assembly. Expression profiling revealed 688 upregulated and 298 downregulated genes in eutopic endometrium compared to controls, while ectopic lesions showed 155 upregulated genes such as Piwi-like RNA-mediated gene silencing 2, fms-related tyrosine kinase 1, and sodium voltage-gated channel alpha subunit 11 [22]. Direct comparison identified 7450 genes overexpressed in ectopic tissue, including oncogenic testis-expressed 41, DNA polymerase theta, and carcinoembryonic antigen-related cell adhesion molecule 1, along with suppression of tumor suppressors such as phospholipase C delta 1 (PLCD1) and odd-skipped related transcription factor 2 (OSR2), underscoring tumor-like properties of invasion and recurrence [23].

Functionally, ectopic lesions are shaped by hypoxia and altered 17β-estradiol (E2) and progesterone (P4) signaling, which reprogram metabolism from oxidative phosphorylation to glycolysis, reminiscent of the Warburg effect in cancer. This shift enhances energy and biomass production, sustaining lesion persistence [24]. Consequently, emerging nonhormonal therapies target these metabolic pathways, including inhibition of glucose transporter type 4 (GLUT4) and regulators of pyruvate metabolism such as lactate dehydrogenase A (LDHA), pyruvate dehydrogenase (PDH), and pyruvate dehydrogenase kinase 1 (PDK1) [25].

Single-cell RNA sequencing has refined this landscape by uncovering the heterogeneity of endometriotic lesions. Ectopic stromal cells preserve endometrial features but aberrantly express genes such as CXCL8, CXCL2, and WNT5A, promoting angiogenesis, wound healing, and inflammation. These cells also interact with ovarian stroma and immune components via Wingless-related integration site (WNT) and bone morphogenetic protein (BMP) signaling, fostering lesion survival [26]. More recent single-cell atlases highlight fibroblasts (FBs) as central to pathogenesis. FBs from ectopic and eutopic sources display distinct but convergent pro-fibrotic, estrogen-driven, and immune-modulatory phenotypes. Aberrant estrogen receptor (ER) β expression, reduced progesterone receptor (PGR), and increased cytochrome P450 family 19 subfamily A member 1 (CYP19A1) disrupt hormone signaling, while FB crosstalks with macrophages, natural killer (NK) cells, and T lymphocytes promotes immune evasion and chronic inflammation. Pathway analysis confirmed activation of transforming growth factor beta (TGF-β), mitogen-activated protein kinase (MAPK), NF-κB, and Janus kinase–signal transducer and activator of transcription (JAK-STAT) signaling, linking FB dysfunction to lesion persistence [27].

Epigenetic alterations further consolidate these molecular programs. Hypermethylation of tumor suppressors such as PGR-B, SF-1, and RASSF1A in ectopic tissue contributes to estrogen dominance and progesterone resistance [28]. Histone modifications regulate genes like PPARγ, HOX10, and ESR1, influencing proliferation, apoptosis, and cell cycle control [29]. In addition, non-coding RNAs, including microRNAs and long non-coding RNAs, fine-tune inflammatory, survival, and immune pathways. Although HOXA10 methylation findings remain variable, histone deacetylase inhibitors (HDACi) have shown potential in suppressing ectopic lesion growth, reinforcing the therapeutic promise of targeting epigenetic mechanisms [21,25].

### 2.2. Disrupted Hormonal Signaling: Local Estrogen Overproduction and Progesterone Resistance

Ectopic endometrial tissue exhibits marked hormonal dysregulation, characterized by excessive local estrogen production and progesterone resistance. Elevated aromatase expression drives abnormal intralesional estrogen accumulation, promoting proliferation and survival, while reduced PGR expression and downstream disruption impair progesterone signaling. This loss weakens anti-inflammatory and pro-apoptotic actions, facilitating lesion persistence. Endometriosis is therefore considered a hormone-dependent disorder sustained by endocrine imbalance and complex molecular mechanisms involving estrogen and progesterone [30].

Estrogens, synthesized from cholesterol, include E2, the most active isoform due to its strong affinity for ERs. ERα and ERβ regulate transcription upon ligand binding, whereas membrane-associated ERs activate rapid PI3K, MAPK, and Ca^2+^ cascades. Dysregulated ER signaling contributes to inflammation and uncontrolled growth. GPER1, a membrane G protein-coupled ER, is aberrantly expressed in ectopic lesions, and its inhibition reduces proliferation and invasion, underscoring its role in progression [31].

Local estrogen production is maintained by aberrant steroidogenesis. Steroidogenic acute regulatory protein (StAR), normally limited to adrenal and gonadal tissues, is overexpressed in stromal cells via prostaglandin E2 (PGE2)-induced cAMP response element-binding protein (CREB) phosphorylation. Aromatase, encoded by CYP19A1, physiologically expressed in granulosa cells, adipose, bone, and brain, is markedly upregulated in endometriosis, especially in ovarian endometriomas with high E2 levels. Enzymes of the 17β-hydroxysteroid dehydrogenase type 1 (HSD17β) family further regulate estrogen activity: the conversion of estrone (E1) to estradiol is increased, while the conversion of E2 to E1 is suppressed [32]. Steroid sulfatase (STS), reactivating estrone sulfate, is upregulated, whereas estrogen sulfotransferase (EST), which inactivates estrogens, is reduced, altogether sustaining estrogen excess [33].

Inflammatory mediators closely regulate these enzymes. PGE2, a potent steroidogenic inducer, activates the cyclic adenosine monophosphate (cAMP)/protein kinase A (PKA) pathway via prostaglandin E2 receptor 2/prostaglandin E2 receptor 4, stimulating steroidogenic factor 1 (SF-1) and CREB to upregulate STAR and CYP19A1 [34]. Cyclooxygenase-2 (COX-2), consistently elevated in stromal cells, drives sustained PGE2 production. Upstream signals such as NF-κB, IL-1β, VEGF, and hypoxia-inducible factor 1 alpha (HIF-1α) amplify COX-2, creating a feedback loop between inflammation and estrogen synthesis. NF-κB also increases cytokine and aromatase expression, reinforcing estrogen dominance [34]. Activated platelets further intensify this loop by engaging NF-κB and transforming growth factor beta 1 (TGF-β1)/mothers against decapentaplegic homolog 3 (Smad3) signaling, which enhances cytokine release, hypoxia, and E2 production through the PGE2-cAMP axis, positioning platelets as amplifiers of estrogen excess.

Progesterone resistance is another hallmark of endometriosis. Ectopic tissue shows reduced PGR expression, particularly loss of PGR-B, disturbing the PRA:PRB ratio and diminishing responsiveness. ERβ overexpression suppresses PR, while PR-B promoter hypermethylation silences this isoform. NF-κB-driven PR-B downregulation and DNA methylation, along with toxicants, genetic variants, and microRNAs (miR-196a, miR-29c, miR-297), further disrupt PGR signaling [35]. Functionally, resistance arises from aberrant PI3K/AKT, MAPK, and Notch receptor 1 (NOTCH1) activation, and impairment of the Indian hedgehog–chicken ovalbumin upstream promoter transcription factor II–Wingless-related integration site 4 signaling cascade (IHH-COUPTFII-WNT4), leading to defective decidualization [36]. Regulators such as signal transducer and activator of transcription 3 (STAT3), B-cell lymphoma 6 (BCL6), and sirtuin 1 (SIRT1) suppress PGR targets, while reduced co-regulators (FK506-binding protein 52, HOXA10, FOXO1, SOX17) weaken progesterone action [29]. Importantly, HSD17β2, normally induced by progesterone to inactivate E2, fails to be expressed, reinforcing estrogen accumulation and progesterone resistance [34].

These hormonal alterations reflect crosstalk between endocrine, inflammatory, and epigenetic pathways. Impaired retinoic acid signaling and PGR downregulation reduce HSD17β2 activity, while a disrupted PR-A/PR-B ratio alters specificity protein 1/specificity protein 3 (Sp1/Sp3)-mediated transcription of enzymes critical for estrogen inactivation [37]. Consequently, estrogen-driven proliferation and survival dominate, while progesterone-mediated differentiation and anti-inflammatory responses remain suppressed.

### 2.3. Key Molecules in the Altered Microenvironment: HIF-1α, VEGF, IL-8, Prostaglandins, and Matrix Metalloproteinases

The microenvironment of ectopic endometrial lesions in endometriosis is shaped by chronic inflammation, dysregulated immune cell activity, increased angiogenesis, hormonal imbalance, hypoxia, oxidative stress, and metabolic alterations [25]. Within this pathological setting, central mediators such as HIF-1α, VEGF, IL-8, prostaglandins, and matrix metalloproteinases (MMPs) sustain lesion growth, proliferation, and invasion. Together, these mechanisms promote lesion persistence and contribute to hallmark symptoms, particularly pelvic pain and infertility [38].

Hypoxia-inducible factor 1 (HIF-1) is a heterodimer composed of the oxygen-regulated HIF-1α and the constitutively expressed HIF-1β subunits [39]. Under low oxygen conditions, HIF-1α stabilizes, translocates to the nucleus, and interacts with HIF-1β to regulate gene transcription via hypoxia-responsive elements. In the endometrium, HIF-1α plays essential roles in tissue repair, decidualization, and maternal–fetal signaling by promoting vascularization and epithelial regeneration through VEGF, IL-8, and adrenomedullin [39]. Dysregulated HIF-1α expression is associated with heavy menstrual bleeding, defective extracellular matrix remodeling, and abnormal neutrophil recruitment [40,41]. In endometriosis, HIF-1α is consistently overexpressed [42], supporting lesion survival through microRNA(miR)-210-3p signaling, mitochondrial adaptation, and autophagy regulation [24,43,44], and facilitating epithelial–mesenchymal transition [45]. Additionally, HIF-1α increases prostacyclin synthase via DNMT1 inhibition, elevating prostacyclin (PGI2) levels and promoting cellular adhesion [46]. It also enhances VEGF, MMPs, IL-8, and COX-2 expression, linking hypoxia to angiogenesis, invasion, prostaglandin production, and inflammation [47].

**Figure 2:**
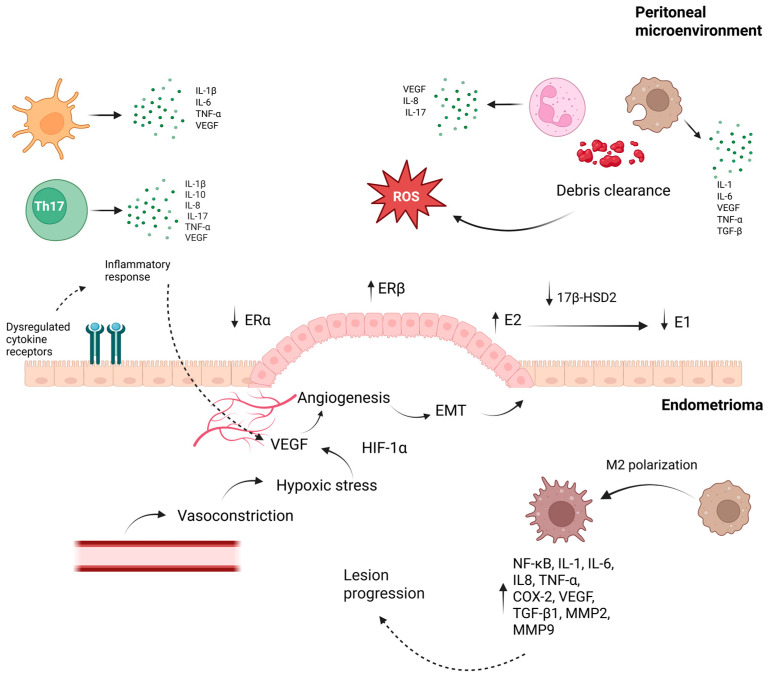
Peritoneal–Endometrioma crosstalk driving inflammation, hypoxia, and lesion progression in endometriosis. The bidirectional interaction shown promotes lesion establishment and progression in endometriosis. In the peritoneal compartment, immune cells release pro-inflammatory cytokines and growth factors—including IL-6, IL-8, IL-17, and VEGF—driving oxidative stress and impairing debris clearance. During menstruation, macrophages and neutrophils clearing endometrial debris generate ROS, and ectopic tissue simultaneously exposed to elevated Fe^2+^, hypoxia, and inflammatory cytokines undergoes oxidative damage, thereby intensifying local inflammatory signaling. Immune dysregulation simultaneously fuels angiogenesis, with macrophages, neutrophils, dendritic cells, and T lymphocytes releasing VEGF together with IL-6, IL-8, and IL-17, a pro-angiogenic effect especially prominent in neutrophils, whose secretion is further intensified by estrogen. Among HIF-1α downstream targets, VEGF is central to angiogenesis, acting through VEGFR-1, VEGFR-2, and VEGFR-3 to promote endothelial proliferation, migration, and vascular permeability. Dysregulated estrogen signaling—characterized by increased ERβ expression and altered local estrogen metabolism via 17β-HSD2—enhances E2 availability, fueling angiogenesis, EMT, oxidative stress, and hypoxia-related responses. Hypoxia-induced VEGF expression, together with vasoconstriction, facilitates neovascularization and supports lesion survival. In parallel, macrophage polarization toward an M2 phenotype maintains a pro-angiogenic and pro-fibrotic milieu through NF-κB activation and the secretion of cytokines, growth factors, and MMPs. In parallel, MMP-2 and MMP-9 function as key downstream effectors regulated by estrogen, inflammatory cytokines, oxidative stress, and PGE2, promoting extracellular matrix degradation, EMT, angiogenesis, and fibrosis; additionally, MMP-9 interacts with PGE2 and TNF-α pathways, reinforcing chronic inflammation and lesion expansion. By enabling endothelial migration and releasing ECM-bound growth factors, MMPs further potentiate VEGF-driven angiogenesis. Together, these interrelated processes establish a self-sustaining inflammatory, estrogen-dependent, and hypoxia-adapted niche that underpins endometrioma persistence and progression [60,61,62,63,64,65]. Abbreviations: Th17: T helper 17 cells; IL: interleukin; TNF-α: tumor necrosis factor alpha; ROS: reactive oxygen species; VEGF: vascular endothelial growth factor; VEGFR: vascular endothelial growth factor receptor; ERα: estrogen receptor alpha; ERβ: estrogen receptor beta; E2: estradiol; E1: estrone; 17β-HSD2: 17 beta-hydroxysteroid dehydrogenase type 2; EMT: epithelial–mesenchymal transition; HIF-1α: hypoxia-inducible factor-1 alpha; NF-κB: nuclear factor kappa B; COX-2: cyclooxygenase-2; TGF-β1: transforming growth factor beta 1; MMP: matrix metalloproteinase; PGE2: prostaglandin E2; ECM: extracellular matrix.arrows; (↑) indicate increased levels or overexpression; (↓) indicate reduced levels or functional downregulation Created in BioRender. Reytor, C. (2026) https://BioRender.com/ndwzjhc (accessed on 12 January 2026).

Elevated VEGF levels in serum and peritoneal fluid of EM patients correlate with lesion vascularization [48,49]. Retrograde menstruation contributes to oxidative stress and cytokine release, promoting VEGF expression, while impaired debris clearance and immune dysregulation further exacerbate lesion progression [50] (Figure 2). Among these immune-derived mediators, IL-8 has emerged as a particularly relevant effector due to its potent angiogenic activity and it is elevated in the peritoneal fluid of EM patients, where it recruits neutrophils and promotes endometrial cell proliferation [51]. Its expression correlates with lesion severity and contributes to neovascularization, nociceptor sensitization, reduced oocyte quality, infertility, and the adhesion, invasion, and proliferation of ectopic endometrial cells [52,53].

Prostaglandins are also critical mediators of the altered microenvironment. Elevated estrogen induces PGE2, which stimulates COX-2 and aromatase, forming a positive feedback loop that sustains hyperestrogenism [30]. In EM, PGE2 reduces macrophage MMP-9 and CD36 expression, impairing phagocytosis and promoting lesion persistence [54]. It further enhances aromatase activity, Th2 polarization, and local estrogen production [55]. PGE2 signaling differs among lesion types, with fibrosis-related variations in COX-2 and 15-hydroxyprostaglandin dehydrogenase shaping disease heterogeneity [56].

MMP expression is elevated in endometriotic tissue, with MMP-9 levels correlating with disease severity [57,58]. Meta-analyses confirm upregulation of MMP-9 in serum and lesions, indicating its potential as a diagnostic biomarker [59].

## 3. Immune-Endometrial Crosstalk: Mechanisms of Escape and Chronicity

### 3.1. Immune Dysfunction and Abnormal Tolerance Toward Ectopic Tissue

Endometriosis is marked by immune dysregulation, with disturbances in both peripheral and endometrial immunity that contribute to infertility, early pregnancy loss, and impaired tissue homeostasis [66]. Clinical evidence shows an increased prevalence of immune-mediated and autoimmune disorders among affected women, suggesting shared pathogenic pathways [67,68,69]. The participation of immune cells is central to disease development, particularly macrophages [70,71], natural killer (NK) cells [72], T lymphocytes [73], and B cells [74]. Hormonal imbalances in endometriosis are known to alter macrophage function, while dysregulated cytokine signaling and impaired immune responses sustain systemic inflammation, a major factor underlying infertility in affected women [75]. Additional immune defects include reduced NK cell cytotoxicity, insufficient dendritic cell maturation, and T cell inhibition through immune checkpoints, all of which establish an immunosuppressive niche that permits lesion survival and progression [75]. Mass cytometry has also demonstrated increased T cell activation in the peritoneal fluid of endometriosis patients compared with controls [76]. More specifically, Huang et al. [77] employed single-cell RNA sequencing of ovarian endometriosis samples and identified five distinct cellular clusters. Among them, mesenchymal endometrial cells predominated and exhibited elevated inflammatory activity and estrogen synthesis. Importantly, the study also revealed a marked reduction in CD8 + T cells within endometriotic lesions, while ectopic T cells displayed diminished cytokine secretion and cytotoxic potential. Such findings highlight CD8 + T cell dysfunction as a critical mechanism fostering abnormal immune tolerance to ectopic endometrial tissue, thereby enabling chronic inflammation and disease persistence.

### 3.2. Roles of NK Cells, M2 Macrophages, Tolerogenic Dendritic Cells, and Treg Cells

Building on the evidence of immune dysregulation in endometriosis, the interplay between innate and adaptive immune cells within the peritoneal cavity further establishes a permissive microenvironment that supports ectopic tissue survival, angiogenesis, and fibrotic remodeling. NK cells exhibit pronounced functional impairment, including reduced expression of granzyme B, perforin, TRAIL, and CD107a, together with increased inhibitory killer immunoglobulin-like receptors, which collectively limit their ability to eliminate refluxed endometrial fragments [65,78]. Concurrently, macrophages are recruited to peritoneal fluid and ectopic lesions, predominantly adopting an M2 (CD206^+^) polarization. These cells contribute to lesion progression by secreting VEGFA, MMP-2 and MMP-9, while impaired phagocytic activity and iron accumulation exacerbate oxidative stress and chronic inflammation [79,80]. Treg cells (CD4^+^CD25^+^FOXP3^+^), which are enriched in peritoneal fluid and lesions, release IL-10 and TGF-β, reinforcing immune tolerance, inhibiting the maturation of imDCs, and indirectly promoting M2 macrophage polarization via FGL2-mediated signaling, thereby establishing a self-sustaining feedback loop between adaptive and innate immunity that supports lesion maintenance [81,82]. Dendritic cells exhibit an altered balance, with increased imDCs (CD1a^+^) and decreased mature DCs (CD83^+^), which facilitate angiogenesis and ectopic tissue survival through the release of IL-10, IL-1β, and IL-6, while MDC1 subsets expressing mannose receptors phagocytose dead stromal cells and amplify local inflammatory signals [83,84,85]. Together, these coordinated cellular alterations consolidate a permissive immune landscape that sustains lesion growth, vascular remodeling, fibrosis, and neurogenic pain.

### 3.3. Immune Evasion Mechanisms: Cytokine Profiles, Integrins, Extracellular Vesicles, Immune Checkpoint Molecules

Beyond cellular composition, immune evasion in endometriosis is reinforced at the molecular level through deregulated cytokine networks, altered adhesion signaling, extracellular vesicle (EV)-mediated communication, and immune checkpoint pathway activation. Elevated concentrations of both pro- and anti-inflammatory cytokines—particularly members of the TGFβ family—are consistently detected in serum, peritoneal fluid, and ectopic lesions, reflecting contributions from epithelial, stromal, mesenchymal, and immune cell populations [86]. Pro-inflammatory mediators and macrophage migration inhibitory factor stimulate angiogenesis, oxidative stress, and aberrant stromal proliferation, while weakening immune cell clearance mechanisms and sustaining chronic inflammation [87,88,89,90,91]. Concurrently, anti-inflammatory cytokines, including IL-4, IL-10, IL-13, IL-33, and IL-37, promote a Th2 and Treg-dominated environment that limits cytotoxic responses and enhances tolerance to ectopic tissue [55,92,93,94]. These cytokine imbalances contribute to ICAM-1 downregulation and abnormal IL-1 family signaling, weakening T-cell interactions with endometrial cells and facilitating lesion persistence through NF-κB activation [95,96].

Aberrant integrin expression further contributes to immune escape and lesion establishment by enhancing adhesion of endometrial cells to peritoneal extracellular matrix components and activating invasive signaling pathways [16,97,98]. Chemokines such as CCL5 reinforce this process by recruiting immune cells and sustaining local inflammation [31]. Meanwhile, EVs derived from immune and endometrial cells serve as potent intercellular messengers that remodel the immune microenvironment. Their molecular cargo, including proteins, miRNAs, and lncRNAs, regulates angiogenesis, immune suppression, and stromal proliferation [86,99]. These vesicles also promote macrophage polarization toward the M2 phenotype through miR-301a-3p and miR-223, suppress T-cell activity via ARG1 induction, and increase extracellular adenosine through CD39/CD73 expression, collectively reinforcing immune tolerance [100,101,102]. Additional EV-mediated signals enhance stromal cell motility and invasion, perpetuating lesion expansion within a “pro-endometriotic niche” [100,102,103,104,105].

Finally, immune checkpoint molecules act as critical modulators of immune suppression in endometriosis. Elevated soluble forms of sPD-L1, sPD-1, sHLA-G, and sCTLA-4 in both serum and peritoneal fluid correlate with lesion severity and infertility [106]. Upregulated PD-1/PD-L1 expression in immune and endometrial cells suppresses T-cell proliferation and cytokine release, reduces NK-cell cytotoxicity, and enhances Treg expansion, leading to immune exhaustion [107,108,109]. Estrogen further induces PD-L1 expression in endometrial epithelial cells, reinforcing local immune tolerance [110]. Overall, cytokine imbalance, altered integrin signaling, EV-mediated suppression, and checkpoint activation collectively sustain an immunosuppressive niche that parallels tumor-like immune evasion [111,112].

### 3.4. Immunomodulatory microRNAs Driving Lesion Persistence

Aberrant regulation of microRNAs (miRNAs) has been identified as a pivotal factor underlying the immune, inflammatory, and hormonal disturbances that sustain lesion persistence and infertility in endometriosis [113,114]. These small non-coding RNAs act as key post-transcriptional regulators of cytokine production, angiogenesis, apoptosis, epithelial–mesenchymal transition (EMT), and progesterone receptor activity, collectively fostering a microenvironment conducive to ectopic tissue survival [115]. Reduced miR-138 activates NF-κB–dependent transcription, enhancing inflammatory and angiogenic mediator expression, while inflammatory stimuli reciprocally induce miR-302a, which suppresses differentiation-related transcription factors and increases cyclooxygenase-2 expression [116]. This bidirectional feedback between miRNAs and cytokines perpetuates chronic inflammation. Downregulation of let-7 family members, particularly let-7b-5p, derepresses ERα, ERβ, aromatase, KRAS, and IL-6, reinforcing estrogen-driven and inflammatory signaling, whereas elevated miR-125b-5p combined with reduced let-7b-5p amplifies macrophage-associated inflammatory outputs [31]. Loss of miR-33b further promotes angiogenesis and proliferation through VEGF and MMP-9 upregulation and reduced apoptotic signaling [47]. Increased miR-146b in peritoneal fluid selectively attenuates M1 macrophage polarization, favoring immune tolerance [117]. Additional miRNAs regulate stromal plasticity and invasiveness. Decreased miR-182 and miR-10b facilitate EMT and IL-6–associated signaling [118], while inflammatory miRNA signatures in peritoneal fluid—including miR-106b-3p, miR-451a, and miR-486-5p—reflect sustained molecular activation within the peritoneal microenvironment [119]. Progesterone resistance is reinforced by miR-29c, miR-135a/b, miR-196a, and miR-194-3p through impaired PR signaling and FKBP4 downregulation [120], while miR-21-5p directly suppresses PR expression, an effect reversible upon its inhibition [121]. Upregulation of miR-29c and miR-143-3p further promotes proliferation, invasion, and EMT through c-Jun and TGF-β/VASH1 pathways [122]. Altogether, dysregulated miRNAs—particularly miR-138, miR-146b, miR-125b-5p, miR-29c, and miR-143-3p—coordinate a network of inflammatory cytokines, angiogenic mediators, and PR pathway suppressors, shaping a chronic, estrogen-dominant, and immune-tolerant environment that perpetuates lesion survival, infertility, and disease progression in endometriosis [117,118,121,122,123,124,125].

## 4. Systemic Footprint: Peripheral Manifestations and Molecular Spillover

### 4.1. Evidence of Low-Grade Systemic Inflammation in Endometriosis Patients

Endometriosis is increasingly recognized as a chronic, low-grade systemic inflammatory disease with physiological repercussions extending far beyond the reproductive tract, such as metabolic, neurological, and immune dysfunction.

Patients frequently display adipocyte and hepatic metabolic disturbances, including reduced body mass index, along with neurobiological changes that heighten pain perception and predispose to fatigue, anxiety, and depression [126]. The systemic inflammatory milieu contributes to localized inflammatory microenvironments in multiple organs and a higher prevalence of immune-mediated diseases [127]. Clinically, endometriosis manifests not only with pelvic pain and infertility but also systemic symptoms such as bowel and bladder dysfunction and persistent fatigue, which affect most patients [128]. Central nervous system involvement is evident, with affected women being almost twice as likely to experience depression compared to controls [129]. Epidemiological studies also indicate elevated cardiovascular risk, with hazard ratios of 1.24 for overall cardiovascular disease and 1.4 for ischemic heart disease [130].

Circulating miRNAs may mediate systemic communication and pathophysiology in endometriosis, as alterations in their abundance are increasingly investigated as diagnostic and mechanistic biomarkers [131,132]. Experimental studies further confirm systemic neurobiological effects: in murine models, endometriosis alters gene expression in brain regions such as the insula, hippocampus, and amygdala, inducing electrophysiological and behavioral changes associated with hyperalgesia, anxiety, and depression [133]. These findings parallel clinical symptoms in women and suggest that neuroinflammation and altered neuronal circuitry contribute to pain sensitization and mood disturbances.

At the molecular level, oxidative stress is pivotal in sustaining chronic inflammation. As seen in Figure 2, the ROS produced creates a redox imbalance that facilitates lesion implantation and correlates with disease severity [134]. Antioxidants such as melatonin and resveratrol demonstrate anti-inflammatory and antioxidative benefits, improving dysmenorrhea and chronic pelvic pain according to meta-analyses. At the systemic level, prostaglandins—particularly PGE2—translate these local inflammatory circuits into sustained pain sensitization and oxidative stress beyond the lesion microenvironment [135]. Proteomic analyses of lesion-like tissue reveal heightened inflammatory and angiogenic signaling, notably through protease-activated receptor pathways. Conversely, reduced expression of the serine protease inhibitor alpha1-antitrypsin augments Toll-like receptor responsiveness, perpetuating chronic inflammation at lesion sites [65,136].

Collectively, this evidence establishes endometriosis as a multisystemic inflammatory condition driven by complex interactions among immune, oxidative, metabolic, and neuroendocrine pathways that reinforce chronic inflammation and systemic dysfunction [135].

### 4.2. Alterations in Peripheral Blood: Soluble Cytokines, microRNAs, Inflammatory Mediators

Consistent with the immune-evasive cytokine networks previously described, endometriosis is associated with reproducible alterations in peripheral blood inflammatory profiles and the peritoneal fluid of affected individuals when compared with healthy controls [137,138]. Elevated IL-1β levels were reported in patients with endometriosis compared with controls [139,140], and a case–control study by Mu et al. [141] confirmed that higher plasma IL-1β levels are associated with an increased likelihood of laparoscopically confirmed endometriosis. Similarly, IL-6 concentrations were found to be significantly higher in women with endometriosis compared to controls [140,142], highlighting its significant role in bridging innate and adaptive immunity through the stimulation of hepatic acute-phase protein synthesis. Higher TNF-α concentrations further support the association between persistent cytokine release and disease maintenance [137,143,144].

These cytokines stimulate hepatic production of acute-phase reactants, including C-reactive protein (CRP). Elevated CRP levels have been reported in women with endometriosis, supporting its potential value as a marker of systemic inflammation [145]. Other inflammatory mediators, including fibrinogen, homocysteine, IL-17, and IL-33, have also been implicated in amplifying this proinflammatory environment [146]. These findings indicate that cytokines and acute-phase reactants derived from endometriotic lesions spread into the systemic circulation, sustaining chronic immune activation [147].

Alterations in microRNA expression further contribute to this inflammatory profile. Benaglia et al. [148] identified increased expression of miR-27b, miR-520f, miR-17, and miR-34a in women with endometriosis, suggesting their role in regulating inflammatory gene networks and immune cell activity. Additional mediators detectable in circulation—including prostaglandins, VEGF, TNF-α, and NGF—reflect molecular spillover from ectopic lesions and contribute to systemic angiogenic and neuroinflammatory signaling [149].

### 4.3. Neuroimmune Links with Chronic Pain, Fatigue, and HPA Axis Dysfunction

Chronic pain represents one of the most disabling manifestations of endometriosis and is driven by profound neuroimmune alterations. In a syngeneic mouse model, Tauseef et al. [150] demonstrated extensive activation of microglia and astrocytes—evidenced by increased IBA1 and GFAP expression in cortical, hippocampal, thalamic, and hypothalamic regions—accompanied by elevated TNF and IL6 levels and behavioral indicators of hyperalgesia and reduced burrowing activity. Hippocampal microglial activation has been implicated in chronic pelvic pain, depression-like behavior, and sex-related differences in mood vulnerability [151,152,153,154], while thalamic microglia respond to systemic stressors and peripheral injury, suggesting that glial dysregulation contributes to central sensitization and emotional disturbances [155]. Clinically, pain—including dysmenorrhea, dyspareunia, and chronic pelvic discomfort—remains the hallmark symptom, often extending beyond the pelvis [132]. Inflammatory mediators sustain nociceptive hypersensitivity and simultaneously modulate central circuits linked to anxiety and depression [156,157]. Elevated IL-6, IL-1β, TNF-α, and CRP levels among women with mood symptoms further support the inflammatory hypothesis of depression [158], wherein cytokines alter neurotransmitter dynamics, neuroendocrine activity, and synaptic plasticity [159].

Fatigue, another disabling feature, may share biological roots with myalgic encephalomyelitis/chronic fatigue syndrome (ME/CFS). Both conditions exhibit persistent immune activation, neuroinflammation, and impaired stress-axis regulation [160,161,162]. The chronic inflammatory milieu in endometriosis establishes systemic biological stress, contributing to central sensitization and neuronal hyperexcitability, which in turn drive widespread hyperalgesia, cognitive dysfunction, and exhaustion [163,164]. Sleep disruption—reported by nearly 70% of affected women—further intensifies inflammation and psychological distress [165], reinforcing this fatigue–inflammation link. Meta-analytic evidence confirms a bidirectional association between endometriosis and ME/CFS, suggesting overlapping neuroimmune pathways [166].

Finally, HPA axis dysregulation serves as a central integrator of these processes. Chronic pain and inflammation alter cortisol secretion patterns and stress responsivity, characteristic of depression and anxiety [167,168]. Peripheral cytokines can cross the blood–brain barrier or signal via vagal afferents, perturbing monoaminergic systems and inducing glucocorticoid resistance and oxidative stress [156,169]. Reduced brain-derived neurotrophic factor (BDNF) levels—associated with impaired neuronal plasticity and emotional regulation—further link inflammation to HPA axis dysfunction and mood pathology, supporting both the inflammatory and neurotrophic hypotheses of depression [170,171].

### 4.4. Emerging Insights into Gut–Endometrium Axis and Microbiome Involvement

The human gut microbiota, often described as a “hidden organ,” constitutes a highly complex community predominantly formed by Firmicutes and Bacteroidetes, alongside Actinobacteria, Proteobacteria, Fusobacteria, and Verrucomicrobia. This microbial ecosystem is essential for nutrient processing, synthesis of bioactive compounds, and the development and regulation of the immune system [172,173]. Early life establishment of commensal microbes is critical for maintaining immune tolerance and homeostasis, yet contemporary lifestyle factors—including industrialization, dietary changes, and decreased microbial exposure—have caused a reduction in microbial diversity and a proliferation of pathogenic species. This dysbiosis compromises immune surveillance and promotes persistent low-grade inflammation [174,175], which is increasingly recognized as a contributor to endometriosis pathophysiology through immune disruption, hormonal imbalance, and systemic inflammatory processes [172,176].

In endometriosis, gut microbiome disturbances are characterized by lower microbial diversity, a higher Firmicutes/Bacteroidetes ratio, increased abundance of pro-inflammatory bacteria such as *Escherichia coli* and *Clostridium*, and a decrease in protective genera like *Lactobacillus* and *Bifidobacterium*, which maintain gut barrier integrity and regulate immune responses [67,177,178,179,180]. Dysbiosis can increase intestinal permeability, allowing translocation of bacterial endotoxins such as lipopolysaccharides (LPS) into circulation. These molecules activate Toll-like receptor pathways, triggering pro-inflammatory signaling and elevating cytokines including IL-6, TNF-α, IL-1β, TGF-β, and IL-18, thereby promoting ectopic lesion formation and sustaining chronic inflammation [173,181]. Gut microbial β-glucuronidase activity, produced by *Bacteroides*, *Bifidobacterium*, *Escherichia*, and *Lactobacillus*, deconjugates estrogens, enhancing their reabsorption and contributing to a hyperestrogenic state that fosters lesion growth [182,183,184,185,186]. Animal studies support these mechanisms: mice with depleted microbiota exhibit reduced lesion growth, whereas overexpression of β-glucuronidase or dysbiosis increases lesion number, size, and macrophage infiltration [187,188]. Stress-induced changes in the gut microbiome further exacerbate inflammatory pathways through the gut–brain axis, amplifying lesion proliferation [189].

Recent studies have highlighted the gut–endometrium axis, whereby microbial metabolites, immune mediators, and estrogen regulation from the gut influence the endometrial microenvironment [190]. Dysbiotic patterns in the endometrial microbiome, including depletion of *Lactobacillus* and overgrowth of *Atopobium*, *Fusobacterium*, and *Gardnerella*, promote inflammation and estrogen-driven proliferation, with implications for estrogen-dependent conditions like endometrial carcinoma [191,192,193,194,195,196]. Functional analyses also indicate alterations in lipid metabolism pathways, such as fatty acid synthase, regulated by estrogen and progesterone, which drive endometrial cell proliferation and may serve as therapeutic targets [197]. Overall, these findings emphasize the multifactorial influence of the gut microbiome on immune modulation, systemic inflammation, hormonal balance, and endometrial tissue regulation, positioning the gut–endometrium axis as a promising focus for microbiome-targeted diagnostics and therapies in endometriosis [198].

## 5. Molecular Biomarkers: Tracking the Dialog

### 5.1. Circulating microRNAs, lncRNAs, and Extracellular Vesicles in Serum and Plasma

Long non-coding RNAs (lncRNAs), which exceed 200 nucleotides and modulate gene expression through chromatin remodeling, RNA stability regulation, and intracellular signaling, have been increasingly detected in circulating blood components of endometriosis patients and are emerging as promising non-invasive biomarkers. Several lncRNAs—including H19, HOTAIR, MALAT1, MEG3-210, and NEAT1—have been reported to show altered expression in serum or plasma, reflecting their involvement in endometriosis pathophysiology [199,200]. Among them, elevated circulating HOTAIR has been associated with increased HDAC1 expression and activation of STAT3-dependent inflammatory pathways through the HOTAIR–miR761–HDAC1 regulatory axis, promoting cytokine-driven inflammation [201]. Network analyses further identified H19 and NEAT1 as central regulatory hubs due to their multiple predicted miRNA binding sites, supporting previous evidence linking these lncRNAs to proliferation and migration of endometrial stromal cells via insulin-like growth factor signaling, with H19 significantly reduced in the eutopic endometrium of affected patients [202,203]. Plasma transcriptome profiling revealed 210 significantly dysregulated lncRNAs in endometriosis, with LINC01569, RP3-399L15.2, FAM138B, and CH507-513H4.6 decreased and RP11-326N17.2, KLHL7-AS1, and MIR548XHG increased compared to healthy controls [204]. Parallel analyses of circulating miRNAs have demonstrated alterations linked to cell migration and proliferation, including reduced serum levels of miR-193 and miR-374 [205]. Extracellular vesicles (EVs) isolated from endometriotic lesions and the peripheral blood of patients carry distinct miRNA signatures, such as let-7a, miR-23a, miR-143, miR-320a, miR-30d-5p, miR-16-5p, miR-27a-3p, and miR-375, which are consistent with their established roles in endometriosis pathogenesis [206,207,208]. Consistently, Ravaggi et al. [209] detected significant upregulation of circulating miR-1249, miR-145-5p, miR-486-5p, miR-485-3p, and miR-26a-5p, alongside downregulation of miR-23a-3p. These findings suggest that dysregulated circulating lncRNAs, miRNAs, and EV-derived miRNAs participate in systemic molecular processes associated with endometriosis, providing a basis for continued exploration of their clinical relevance.

### 5.2. Peritoneal Fluid Proteomics and Systemic Inflammatory Profiles

Proteomic investigations of both peritoneal fluid and peripheral blood demonstrate distinct immune-inflammatory disturbances in women with endometriosis, indicating that the condition is associated with both localized and systemic activation of pathological pathways. Elevated levels of key inflammatory mediators, including interleukin-1β (IL-1β), interleukin-6 (IL-6), tumor necrosis factor-α (TNF-α), and C-reactive protein (CRP), have been consistently observed in affected individuals when compared with controls, supporting the presence of a heightened inflammatory state that is not restricted to the peritoneal cavity [76,210,211,212]. In a comprehensive plasma proteomic analysis, Sasamoto et al. [210] reported increased expression of proteins associated with immune activation, oxidative stress, iron regulation, and angiogenesis, including protein kinase C zeta type, ferritin, hepcidin, peroxiredoxin-6, ephrin-B3, angiopoietin-related protein 3, RNA-binding protein 39, gremlin-1, hepatocyte growth factor, and cathepsin G. Among these, ferritin and hepcidin were prominently upregulated, implicating altered systemic iron homeostasis, while angiogenesis-related factors such as angiopoietin-related protein 3, gremlin-1, and hepatocyte growth factor underscored the role of vascular remodeling in disease progression. Parallel analysis of peritoneal fluid, which provides direct insight into the local environment of endometriotic lesions, has further illuminated disease-associated proteomic alterations. Janša et al. [213] identified 16 proteins with differential abundance in endometriosis compared to infertility controls, including the proinflammatory calcium-binding proteins S100A8/9, scavenger receptor cysteine-rich type 1 protein M130, Epidermal Growth Factor Receptor (EGFR), and Tissue Inhibitor of Metalloproteinase 1 (TIMP1). These findings reflect an integrative network of inflammatory and proteomic alterations that shape the pathophysiological environment in endometriosis.

### 5.3. Integration of Omics-Based Biomarkers for Early Diagnosis and Patient Stratification

The integration of multi-omics technologies is emerging as a transformative approach for the early diagnosis and clinical stratification of endometriosis, addressing current limitations related to delayed detection and heterogeneous clinical presentation. Genomic studies have identified susceptibility loci such as *WNT4*, *PGR* and *GREB1*, enabling the development of polygenic risk scores that may predict disease predisposition [214], while epigenomic analyses have revealed aberrant DNA methylation and dysregulated non-coding RNAs that contribute to altered hormonal responses, inflammation, and aberrant cell proliferation [28,115,215]. Transcriptomic profiling, including circulating microRNAs and long non-coding RNAs in plasma, endometrial tissue and peritoneal fluid, has demonstrated substantial diagnostic potential due to their stability and disease-specific expression patterns [216]. Proteomic analyses further complement these findings by identifying dysregulated proteins in serum and peritoneal fluid related to immune activation, angiogenesis and extracellular matrix remodeling, including alterations in cytokines, tumor necrosis factor signaling pathways, S100 proteins, and TIMP family members [217]. Likewise, metabolomic studies have characterized distinct metabolic fingerprints associated with oxidative stress, estrogen metabolism and immune dysregulation, suggesting their utility in distinguishing endometriosis from other gynecologic pathologies [218]. The integration of these omics layers through systems biology and network-based analytical approaches allows for the identification of biomarker panels rather than isolated markers, enhancing diagnostic sensitivity and specificity [219,220]. Machine learning and artificial intelligence are increasingly employed to combine genomic, proteomic, metabolomic and microbiomic datasets, enabling the classification of patients into biologically meaningful subgroups, such as inflammatory-dominant, fibrotic or hormone-resistant phenotypes [221]. This stratification has critical implications for predicting disease progression, assessing infertility risk and guiding personalized therapeutic strategies. Importantly, the incorporation of microbiome data, particularly alterations in the gut and reproductive tract microbial communities, further refines patient profiling and may offer novel non-invasive diagnostic tools [222,223]. While the clinical translation of multi-omics biomarkers is still challenged by variability in sample collection protocols and the need for validation in large cohorts [224], ongoing advances in integrative platforms and liquid biopsy technologies are paving the way for dynamic biomarker models capable of monitoring disease activity and recurrence in real time [225,226]. Collectively, multi-omics integration represents a fundamental shift toward precision medicine in endometriosis, offering the potential to revolutionize disease detection, classification and individualized treatment selection.

## 6. Therapeutic Opportunities in a Systemic Disease

### 6.1. Limitations of Conventional Hormonal Therapies

Hormonal therapy continues to play a central role in the management of endometriosis, though its long-term effectiveness and patient adherence are constrained by several limitations. Progestins, such as dienogest, norethindrone acetate, medroxyprogesterone acetate, cyproterone acetate, Implanon, and levonorgestrel-releasing intrauterine systems, act by suppressing gonadotropin secretion, inducing decidualization and endometrial atrophy, and modulating local immune-inflammatory responses, including decreases in IL-6, IL-8, MCP-1, and TNF-α-driven cell proliferation [227,228]. Nevertheless, approximately one-third of patients experience progesterone resistance, commonly linked to reduced expression of progesterone receptors (PGR) in endometriotic tissue, which substantially reduces the therapeutic effect of progestins [229]. Dienogest has demonstrated clinical benefits in alleviating pain and reducing lesion size, even in deep infiltrating endometriosis and adenomyosis, though its use can be complicated by initial bleeding irregularities and temporary decreases in bone mineral density, especially in adolescents treated for over one year [230,231]. Other progestins often require higher, sometimes unapproved, doses to achieve comparable effects, while depot formulations like MPA or norethisterone enanthate, although efficacious, are associated with a higher risk of adverse events such as thrombosis, bone loss, and systemic hypoestrogenic symptoms [232].

Combined oral contraceptives (COCs) are effective in controlling dysmenorrhea by inhibiting ovulation and stabilizing the endometrium, yet their impact on non-menstrual pelvic pain or dyspareunia is limited, and early use has been associated with increased risk of deep infiltrating endometriosis [233]. Extended-cycle regimens, vaginal rings, and other combined estrogen–progestin preparations are generally well-tolerated, but limitations remain due to contraindications linked to the estrogen component and incomplete symptom control [228]. The LNG-IUS provides targeted therapy with minimal systemic side effects and sustained lesion suppression, particularly after surgery for rectovaginal endometriosis or adenomyosis; however, it is not FDA-approved for endometriosis-related pain and discontinuation may occur due to irregular bleeding, depression, or weight gain [234].

GnRH agonists and antagonists efficiently reduce estrogen levels and lesion stimulation, but their utility is limited by pronounced hypoestrogenic effects—hot flashes, sleep disturbances, vaginal dryness, mood changes, bone loss—and high recurrence rates, with up to 50% of patients experiencing symptom relapse within six months of therapy cessation [235]. Emerging therapies, including linzagolix and relugolix combination therapy, have demonstrated rapid and sustained improvement in dysmenorrhea, non-menstrual pelvic pain, and dyspareunia, while add-back therapy helps mitigate hypoestrogenic side effects; however, long-term safety, adherence, and cost considerations require further study [236,237,238]. Across all hormonal options, common adverse effects—bloating, weight gain, acne, breast tenderness, libido changes, irregular bleeding, fatigue, and, less frequently, mood disturbances—pose challenges for adherence, emphasizing the importance of individualized treatment planning [239].

Finally, endometriosis recurrence, ranging from 10% to 80% depending on surgical completeness and disease severity, highlights the need to integrate pharmacological and surgical interventions, as monotherapy rarely eradicates microscopic disease [233]. While hormonal treatments offer substantial symptom relief and improved quality of life, their variable efficacy, progesterone resistance, hypoestrogenic side effects, bleeding irregularities, and risk of recurrence necessitate careful monitoring, patient counseling, and tailored management strategies to optimize outcomes.

### 6.2. Promising Molecular Targets: Angiogenesis Inhibitors, Immune Modulators, Epigenetic Regulators

Current research aims to identify drugs that selectively modulate the hormonal and immunological microenvironment of endometriotic lesions to suppress proliferation, promote apoptosis, and normalize aberrant invasion and angiogenesis (Table 1) [240]. Among angiogenesis-targeted therapies, VEGF is a central mediator, with elevated levels observed in peritoneal fluid and tissue, making it a primary target [241,242]. The MMP complex—including fibroblast growth factor, MMP2, and MMP9—also contributes to invasion and metastatic-like dissemination of endometrial cells and is considered a therapeutic target [243]. Dopamine and dopamine receptor-2 agonists reduce neoangiogenesis by promoting VEGF receptor endocytosis, supporting their potential as antiangiogenic agents [244,245]. Similarly, RAS pathway inhibitors such as ACEIs and ARBs attenuate VEGF-mediated angiogenesis, suppress inflammatory cytokines including IL-6 and TNF-α, and reduce fibrosis and adhesion formation, potentially improving reproductive outcomes [246,247].

Epigenetic regulators are also key targets, as chronic inflammation induces DNA hypermethylation affecting HOXA10, a transcription factor linked to endometrial receptivity and progesterone receptor expression [248]. The DNA methylation inhibitor 5′-aza-deoxycytidine (AZA) reverses HOXA10 hypermethylation in vitro, providing mechanistic support for future therapies [249], although currently approved agents remain toxic for fertility-seeking patients [248]. Progesterone receptor gene methylation further contributes to aberrant silencing, highlighting the potential of demethylating agents and histone deacetylase inhibitors [250].

Elevated ROS due to impaired detoxification pathways sustains proliferative signaling [251], while proliferative cascades such as RAF/MEK/ERK and PI3K/Akt/mTOR can be inhibited by cannabinoid agonists in deep nodular lesions, underscoring the convergence of multiple growth-promoting pathways [247]. EMT contributes to invasive phenotypes in ovarian, peritoneal, and deep-infiltrating endometriosis, with partial EMT confirmed by Konrad et al. [252] and estrogen-associated EMT markers supported by Liu et al. [253], suggesting pharmacological inhibition of EMT regulators as a potential strategy.

Autophagy-apoptosis pathways represent additional targets; silencing cirRNA promotes apoptosis of ectopic endometrial cells, and SCM-198, a synthetic pseudoalkaloid, reverses a hypo-autophagic state induced by increased ERα and decreased PR-B expression, restoring autophagy and promoting apoptosis [254].

Targeting inflammatory and immune pathways remains critical due to the overproduction of prostaglandins, cytokines, and other mediators. Nonselective NSAIDs inhibit COX-1 and COX-2 and provide symptomatic relief, while TNF-α inhibitors have been explored for disease modulation [255]. Aberrant immune regulation, including NK cell inhibition and macrophage hyperactivation, represents actionable targets [256,257,258], and PD-1/PD-L1 immune checkpoint inhibitors may restore immune tolerance and enhance anti-endometriotic responses [259,260,261]. TGF-β, which suppresses NK cell cytotoxicity and other immune functions, is another potential target [262,263]. Furthermore, strategies restoring immune surveillance and modulating ribosome biosynthesis (affecting macrophage proliferation via mTOR/PI3K and RNA polymerase I inhibition) have demonstrated anti-inflammatory and analgesic effects in preclinical models [247,264].

Finally, RAS-targeting compounds also constitute an avenue for continued investigation and development of novel therapeutic approaches for endometriosis. These agents act on key mechanisms of disease progression—angiogenesis, inflammation, and fibrosis—thereby limiting lesion growth, reducing pain, and minimizing adhesion formation, which underscores their promise as non-hormonal therapeutic options for endometriosis management [246].

**Table 1:** Agents Investigated as Inhibitors of Molecular Pathways in Endometriosis. This table lists agents, their molecular targets, and the pathways through which they modulate inflammation, cell proliferation, migration, and apoptosis in endometriotic lesions. Abbreviations: IL: Interleukin; MCP-1: Monocyte Chemoattractant Protein-1; NF-κB/NF-kB: Nuclear Factor kappa-light-chain-enhancer of activated B cells; TNF-α: Tumor Necrosis Factor-alpha; mRNA: Messenger Ribonucleic Acid; VEGF: Vascular Endothelial Growth Factor; VEGFR2: Vascular Endothelial Growth Factor Receptor 2; E-cadherin: Epithelial cadherin; N-cadherin: Neural cadherin.

Inhibitor	Target Proteins	Mechanism of Action
Resveratrol [265]	IL-6, IL-1B, MCP-1	Reduces the expression of inflammatory markers in the eutopic endometrium and, to a greater extent, in the ectopic endometrium.
Curcumin [266]	NF-kB, cytokine production	By inhibiting NF-κB signaling and lowering cytokine levels, preclinical studies indicate its potential to reduce lesion size and associated pain.
Genistein [267]	NF-kB, TNF- α, IL-6, IL-8	Suppresses the expression of inflammatory mediators and reduces cell proliferation.
Leflunomide [248]	NF-kB	Inhibits proliferative activity in endometrial cells.
Nobiletin [268]	NF-kB, IL-6, IL-1B	Mitigates lesion growth and pain through inhibition of cell proliferation, angiogenesis, and excessive inflammation.
Ginsenoside [269]	NF-kB, protein kinase B	Limits endometrial stromal cell viability via inhibition of NF-κB signaling.
Dienogest [270]	NF-kB, TNF- α, IL-8	Reduces IL-8 expression by inhibiting TNF-α–mediated activation of NF-κB.
Thalidomide [271]	NF-kB, TNF- α, IL-8	Reduces IL-8 expression at both the microRNA and protein levels by inhibiting TNF-α–induced NF-κB activation.
Tocilizumab [272]	IL-6	Monoclonal anti-IL-6 antibody demonstrated to induce regression of lesions.
Quinagolide [273]	VEGF/VEGFR2 pathway	Demonstrated to markedly reduce lesion size, possibly through modulation of angiogenesis.
Fucoidan [274]	Snail and Slug, Notch	Inhibits the growth of endometriotic lesions and triggers apoptosis through anti-proliferative and anti-inflammatory mechanisms.
Pyrvinum pamoate [275]	IL-6, IL-8	Downregulates IL-6 and IL-8 mRNA expression.
Isoliquiritigenin [276]	Snail, Slug, E-cadherin, N-cadherin	Attenuates the inflammatory response while inducing apoptosis.
3,6-dihydroxyflavone [277]	Notch signaling pathway	Inhibits cell migration through downregulation of Notch and associated downstream molecules.
Melatonin [278]	Snail, Slug, Notch homolog, E-cadherin, N-cadherin	Reduces endometriotic lesion growth and invasiveness by inhibiting estradiol and Notch signaling.

### 6.3. Advances in Targeted Delivery Systems and Nanotherapeutics

Current therapeutic advances in endometriosis are increasingly directed toward targeting dysregulated molecular and immune pathways rather than relying solely on hormonal suppression. Regulatory miRNAs such as let-7b, miR-125, and miR-155 modulate lymphocyte activity and inflammatory signaling, with let-7b shown to reduce lesion size in macaque models [243]. Similarly, bentamapimod, a c-Jun N-terminal kinase inhibitor, promotes lesion regression without affecting menstrual cyclicity. Other promising strategies include targeting MAPK/ERK, NF-κB, HIF-1α, and MMP-2/-9, which are central to cell proliferation, invasion, and angiogenesis [279]. Immunomodulatory approaches, including TNF-α inhibitors, immune checkpoint blockade, siRNAs, and miRNA mimics, are advancing through preclinical development and may enable precision medicine guided by molecular biomarkers [175,248,280].

As part of this emerging therapeutic landscape, nanotechnology offers one of the most innovative and clinically promising strategies, enabling selective delivery of therapeutic agents directly to endometriotic lesions [281,282]. Nanoparticles accumulate at disease sites via the enhanced permeability and retention effect and can be engineered to actively target receptors such as VEGFR2 (KDR) and EphB4, which are overexpressed in endometrial tissue [280]. Polymeric nanocarriers like Poly(ethylene glycol)-poly(ε-caprolactone) facilitate controlled drug release, while magnetic nanoparticles like KDR-Manganese-containing nanoparticles combine imaging capabilities with magnetothermal therapy to destroy lesions. Gold nanoshells such as hollow gold nanoshells (HAuNS) and its derivates utilize near-infrared light to generate localized heat and induce apoptosis of endometriotic cells [283]. Small interfering RNA-loaded nanoparticles paired with cell-penetrating peptides enable gene-targeted interventions that suppress key pathogenic pathways [284,285]. Theranostic platforms, such as SiNc-PEG-PCL nanoparticles, allow simultaneous lesion detection and photothermal ablation, while Poly(lactic-co-glycolic) acid -based systems co-loaded with doxycycline and Epigallocatechin-3-gallate inhibit angiogenesis and oxidative stress in animal models [240]. Incorporating agents like danazol or Elagolix into nanoparticle formulations further enhances drug bioavailability and reduces systemic toxicity [280]. Although additional clinical validation is required, nanotherapeutics represent a significant step toward highly targeted, effective, and personalized treatment strategies for endometriosis [283].

### 6.4. Potential of Immunotherapy and Early Systemic Intervention

Emerging strategies for immunotherapy and early systemic intervention in endometriosis have highlighted mesenchymal stem cells (MSCs), particularly endometrial MSCs (eMSCs), due to their self-renewal, multipotency, and potent immunomodulatory properties [286,287]. MSCs can be isolated from bone marrow, adipose tissue, placenta, and endometrium, with eMSCs being especially relevant given their physiological role in endometrial regeneration and tissue homeostasis [288,289]. In the context of endometriosis, however, MSCs derived from ectopic lesions exhibit altered phenotypes, including upregulation of inflammatory (IL-6, TNF-α) and angiogenic (VEGF) genes, enhanced proliferation and migration, and dysregulated immune interactions, potentially facilitating ectopic implantation and contributing to ovarian carcinogenesis [286,289,290]. Mechanistically, MSCs exert immunomodulatory effects through exosome-mediated delivery of cytokines and miRNAs, which suppress effector T cells and pro-inflammatory macrophages while promoting regulatory cells such as B regs, thereby restoring immune homeostasis [291,292]. Moreover, MSCs inhibit angiogenesis via the miRNA-21-5p/TIMP3/PI3K/Akt/mTOR pathway and downregulation of VEGF, while simultaneously promoting endometrial repair and improving fertility outcomes [4,288,290,292,293]. Preclinical models demonstrate reductions in lesion size, inflammatory cytokines, and angiogenic markers following MSC therapy [286,289]. Although initial clinical reports suggest improvements in fertility and symptom relief, further optimization of MSC source, administration protocols, long-term safety, and development of exosome-based or genetically modified approaches are required to maximize therapeutic efficacy.

## 7. The Case for a Systems Medicine Approach

### 7.1. Proposal to Reconceptualize Endometriosis as a Systemic Inflammatory Disorder with Gynecologic Expression

Growing evidence supports a shift toward viewing endometriosis as a systemic inflammatory condition, in which gynecologic lesions represent one aspect of a broader, immune-mediated pathology [147,294]. Rather than being confined to pelvic sites, the condition induces body-wide inflammatory activity, demonstrated by elevated serum concentrations of TNF-α, IL-1β, and IL-6 [119], alongside profound alterations in both local and systemic immune responses [295]. Circulating microRNAs further contribute to this systemic inflammatory cascade; specifically, dysregulation of miRNA 125b-5p and Let-7b-5p promotes enhanced cytokine production by macrophages at distant sites, indicating communication between lesions and peripheral immune compartments via molecular signaling networks [119]. The systemic nature of the disease is also reflected in metabolic disturbances, including altered adipocyte and hepatic function associated with low body mass index, widespread neuroimmune alterations driving central sensitization and mood disorders such as fatigue, anxiety, and depression [296], chronic inflammatory-like conditions in non-reproductive tissues [297], and a predisposition to immune-mediated diseases [127]. Furthermore, systemic inflammation in endometriosis is linked with increased cardiovascular risk, malignancy, and a hypercoagulable state [147,298]. Extra-pelvic manifestations, including thoracic endometriosis, undermine the idea that retrograde menstruation alone accounts for disease development and instead point to mechanisms such as cellular dissemination and immune system activation [299]. These complex, system-wide effects, together with high comorbidity rates and persistent lack of a definitive cure, emphasize that endometriosis is fundamentally a systemic condition requiring integrated therapeutic strategies involving multiple specialties rather than exclusively localized surgical management. Reframing the disease in this way provides a foundation for identifying novel molecular targets and developing treatments that modulate systemic inflammatory and immune pathways rather than addressing only local manifestations [294].

Clinically, reframing endometriosis as a systemic inflammatory disorder has direct implications for diagnostic pathways. This perspective supports the incorporation of circulating molecular markers—including cytokines, microRNAs, extracellular vesicles, and proteomic signatures—into non-invasive diagnostic strategies to complement or potentially reduce reliance on surgical confirmation [294]. In addition to pelvic pain and infertility, clinical suspicion should encompass systemic manifestations such as fatigue, mood alterations, metabolic disturbances, and cardiovascular risk indicators, thereby broadening diagnostic vigilance beyond gynecologic symptoms alone. Multidisciplinary assessment involving gynecology, immunology, neurology, cardiology, and mental health specialists may therefore be warranted in selected patients, particularly those exhibiting significant systemic involvement [147]. Such an approach aligns with emerging evidence that early recognition of systemic disease features may enable earlier diagnosis, improved monitoring, and more comprehensive patient care.

### 7.2. Systems Biology Tools: Network-Based Analyses, Transcriptomics, Proteomics, and Integrative Multi-Omics

Systems biology approaches have become pivotal in advancing the understanding of endometriosis, a heterogeneous and multifactorial disorder characterized by complex interactions between hormonal signaling, immune dysregulation, inflammatory mediators, stromal remodeling, angiogenesis, and neurogenesis [300]. Unlike traditional reductionist analyses that focus on single genes or pathways, system-level methodologies enable the identification of interconnected molecular networks and disease-specific patterns that underlie lesion establishment, chronic inflammation, and symptom heterogeneity [301]. Network-based analyses, including gene co-expression networks and protein–protein interaction models, have revealed central hub genes and signaling pathways that act as regulatory drivers of the disease, such as the PI3K/AKT/mTOR, NF-κB, and Wnt/β-catenin pathways [302]. These analyses have also demonstrated molecular convergence between endometriosis and other chronic inflammatory or autoimmune conditions, highlighting shared pathogenic mechanisms and suggesting potential opportunities for therapeutic repurposing [303,304].

Transcriptomic profiling using microarrays and RNA sequencing has revealed distinct gene expression signatures in both eutopic and ectopic endometrium, including dysregulation of genes associated with extracellular matrix remodeling, immune activation, angiogenesis, hypoxia response, and neural infiltration [305]. Importantly, transcriptomic studies have identified molecular endotypes that differentiate lesion subtypes and disease stages, providing insight into inter-individual variability in symptom severity and treatment response [306]. Proteomic analyses have further expanded this understanding by identifying alterations in the protein landscape of endometrial tissue, peritoneal fluid, and circulating blood, particularly involving cytokines, chemokines, adhesion molecules, and matrix metalloproteinases [213]. These protein-level changes contribute to lesion survival, immune evasion, and nociceptive signaling, while also providing promising biomarker candidates for non-invasive diagnostics.

The integration of multi-omics approaches—combining genomics, epigenomics, transcriptomics, proteomics, metabolomics, and microbiome data—has allowed the construction of comprehensive molecular profiles that capture the dynamic interplay between genetic predisposition and environmental factors [307,308]. Multi-omics integration using network-based algorithms and machine learning techniques enables the stratification of patients into molecular subgroups, facilitates the prediction of therapeutic responsiveness, and identifies novel targets for precision medicine [309]. Together, systems biology tools are transforming endometriosis research by elucidating disease mechanisms at a network level, uncovering novel biomarkers, and paving the way toward personalized therapeutic strategies.

Beyond mechanistic insight, systems biology approaches provide an opportunity to develop clinically meaningful patient stratification frameworks. Integrative multi-omics analyses increasingly support the existence of distinct biological endotypes, such as inflammation-dominant, immune-dysregulated, hormone-resistant, angiogenesis-driven, neuroimmune-pain-dominant, and fibrosis-predominant phenotypes [111,145,219]. These subgroups may differ in symptom profiles, disease progression, comorbidity risk, and therapeutic responsiveness, highlighting the need for tailored management rather than uniform treatment strategies. Stratification based on molecular signatures, combined with clinical features and imaging findings, holds potential to guide prognosis estimation, refine therapeutic decision-making, and support the design of precision-based clinical trials that better capture disease heterogeneity [219].

### 7.3. Pathway Toward Precision Medicine: Tailoring Treatments Based on Molecular Endotypes

Over the last twenty years, the therapeutic landscape of endometriosis has evolved from generalized hormonal suppression and surgical excision to advanced approaches that prioritize individualized treatment based on the molecular and cellular characteristics of the disease [310]. This transformation is grounded in the recognition that endometriosis is not a uniform condition, but rather a collection of biologically distinct endotypes characterized by unique genetic, immunological, and epigenetic profiles that dictate how patients respond to therapy [311,312,313]. In this emerging framework of precision medicine, three innovative strategies have gained prominence: targeted molecular agents, nanoparticle-mediated drug delivery systems, and stem cell–based regenerative therapies. Targeted therapies aim to selectively inhibit aberrant signaling pathways implicated in lesion survival and inflammation, thereby offering higher specificity and reduced systemic side effects compared to traditional hormonal interventions [282]. Nanoparticles further enhance therapeutic precision by enabling localized drug delivery to endometriotic lesions and improving pharmacokinetic properties, while also serving as carriers for bioactive molecules secreted by stem cells [314,315]. Stem cell therapies introduce the possibility of not only suppressing pathological processes but also restoring normal tissue architecture and modulating immune dysregulation [316]. Importantly, these therapeutic modalities are not isolated in their application; rather, they may be combined in synergistic regimens—for instance, nanoparticles can be engineered to carry targeted drugs or stem cell–derived factors, thereby amplifying treatment efficacy and potentially decreasing the need for surgical intervention [317]. Such integrative, biology-driven strategies represent a model for truly personalized care, where treatment is precisely aligned with a patient’s unique molecular endotype [282].

From a therapeutic standpoint, recognition of molecular endotypes provides a framework for aligning treatment selection with underlying disease biology. Patients with inflammation- or immune-dominant profiles may benefit most from immunomodulatory or anti-inflammatory targeted agents, whereas angiogenic or fibrotic phenotypes may be more responsive to anti-angiogenic or anti-fibrotic approaches and regenerative strategies [93]. Hormone-resistant endotypes may require alternatives beyond conventional endocrine suppression, including pathway-targeted therapies or epigenetic modulation [35]. Importantly, treatment considerations should extend beyond lesion burden to include systemic inflammatory load and comorbidity risk, reinforcing the rationale for systemic therapeutic strategies in appropriate patients. Biomarker-guided therapeutic allocation, supported by systems biology–derived molecular signatures, may therefore enhance efficacy, reduce overtreatment, and advance the implementation of precision medicine in routine clinical practice.

Future research must focus on several critical fronts: conducting large-scale phase III clinical trials to evaluate targeted therapies and nanotechnology-based treatments; advancing translational studies to establish the safety and therapeutic viability of stem cell applications; and identifying predictive and prognostic biomarkers that can stratify patients and guide individualized treatment decisions. Additionally, investigating combination approaches will help determine how different therapeutic platforms can be optimally integrated to achieve synergistic effects, while the development of gene and epigenetic therapies—particularly those utilizing CRISPR and siRNA technologies—holds promise for directly correcting pathogenic mechanisms at the molecular level [282]. Collectively, these advances delineate a clear pathway toward precision medicine in endometriosis, shifting the paradigm from symptom management to mechanism-based intervention tailored to the biological drivers of disease in each patient.

### 7.4. Clinical and Translational Implications

Recognizing endometriosis as a systemic disorder has meaningful clinical consequences, supporting broader diagnostic consideration, incorporation of circulating biomarkers, and movement toward molecularly informed therapeutic strategies. Systems biology and multi-omics approaches offer the potential to stratify patients into biologically meaningful endotypes, refine prognosis, and guide personalized treatment allocation, while advances in targeted agents, immunomodulatory therapies, nanotechnology-based delivery systems, and regenerative approaches provide promising future avenues [315]. Together, these developments outline a framework in which diagnostic workflows, clinical decision-making, and long-term disease management may increasingly shift from lesion-centered care toward precision medicine paradigms.

Nevertheless, interpretation of the mechanistic evidence synthesized in this review requires careful consideration of the marked clinical and biological heterogeneity of endometriosis. The studies discussed encompass diverse disease phenotypes, including ovarian, peritoneal, and deep infiltrating endometriosis, which may differ substantially in molecular signatures, inflammatory profiles, and pathogenic drivers. As a result, the integration of findings across these entities may introduce confounding effects when extrapolating shared mechanisms.

Moreover, it is essential to emphasize that the clinical translation of these advances remains at an early stage [314]. Much of the current evidence derives from preclinical studies, exploratory biomarker research, or small and heterogeneous cohorts, which limits generalizability and prevents firm conclusions regarding clinical utility. In addition, patients included across studies are unlikely to share uniform etiological pathways or disease trajectories, as endometriosis can arise through multiple, potentially overlapping mechanisms (i.e., retrograde menstruation, developmental Müllerian anomalies, uterine tract abnormalities), as well as varying disease stages and lesion burdens. This intrinsic variability represents an important confounding factor and underscores the need for caution when drawing unifying mechanistic conclusions.

Beyond its gynecological manifestations, growing epidemiological and molecular evidence indicates an association between endometriosis and an increased risk of certain malignancies, most notably ovarian clear cell and endometrioid carcinomas [290,318]. This link has stimulated interest in shared biological processes that may underlie both conditions, including chronic inflammation, estrogen-driven signaling, immune dysregulation, oxidative stress, aberrant epigenetic modifications, and sustained activation of oncogenic pathways such as PI3K/AKT, MAPK, and Wnt/β-catenin [318]. In support of this overlap, somatic mutations commonly identified in endometriosis-associated lesions—such as ARID1A, PIK3CA, and KRAS—mirror alterations frequently observed in endometriosis-associated ovarian cancers, suggesting that a subset of lesions may acquire cancer-like molecular features without inevitably progressing to malignancy [319]. Importantly, endometriosis should not be considered a premalignant disease; however, these shared molecular vulnerabilities may contribute to lesion persistence, therapeutic resistance, and, in specific biological contexts, malignant transformation [6]. From a translational perspective, this convergence underscores the potential value of biomarker-driven risk stratification, targeted therapeutic strategies, and tailored long-term surveillance in selected patient subgroups, while acknowledging that the absolute cancer risk remains low and causality is not fully established.

Multi-omics platforms and AI-driven models require methodological standardization, reproducibility across populations, rigorous external validation, and integration into clinically feasible workflows before they can be implemented reliably [320]. Moreover, realistic adoption is challenged by economic burden, infrastructure requirements, unequal accessibility across healthcare systems, ethical and regulatory considerations, and the need for clinician training and health-policy adaptation [321]. Future translational progress will therefore depend on well-powered, stratified clinical studies that explicitly account for disease subtype, stage, and pathogenic context, enabling more precise linkage between molecular mechanisms and clinically relevant endotypes. Therefore, while these strategies hold substantial promise, their incorporation into routine practice will depend on large, well-designed clinical trials, cost-effectiveness evaluation, standardized protocols, and equitable implementation frameworks.

## 8. Conclusions

Emerging evidence increasingly positions endometriosis as a complex systemic disorder rather than a condition confined to pelvic lesions. This reconceptualization reflects the multifactorial and chronic nature of the disease, characterized by intricate molecular interactions between ectopic endometrial tissue, the immune system, and peripheral organs. Ectopic endometrium exhibits unique transcriptomic and epigenetic signatures alongside disrupted hormonal signaling, including local estrogen overproduction and progesterone resistance. These molecular aberrations establish a pro-inflammatory microenvironment enriched with cytokines, prostaglandins, matrix metalloproteinases, and angiogenic factors such as VEGF and HIF-1α, which collectively facilitate lesion survival, proliferation, and invasiveness. Such evidence challenges the sufficiency of lesion-centric models and highlights the importance of framing endometriosis within a systemic and molecular context.

Central to disease chronicity is immune-endometrial crosstalk. Dysregulation of innate and adaptive immune components—including NK cells, M2 macrophages, tolerogenic dendritic cells, and regulatory T cells—enables ectopic tissue to escape immune surveillance. Immune evasion is reinforced by altered cytokine environments, integrin-mediated interactions, extracellular vesicles, immune checkpoint molecules, and immunomodulatory microRNAs, collectively sustaining lesion persistence and propagating inflammation. This interplay between immune dysfunction and molecular dysregulation underscores how localized lesions drive systemic immune perturbations and contribute to the heterogeneity of clinical manifestations among patients.

Beyond the pelvic cavity, endometriosis exerts a measurable systemic footprint. Peripheral low-grade inflammation, circulating cytokines, microRNAs, and other inflammatory mediators reflect molecular spillover from ectopic tissue. Neuroimmune pathways link these systemic signals to chronic pain, fatigue, and hypothalamic–pituitary–adrenal axis dysregulation, while emerging research implicates gut–endometrium interactions and microbiome alterations in modulating disease expression. These observations reinforce the notion that endometriosis should be approached as a systemic disorder with multisystem involvement, necessitating integrated diagnostic and therapeutic strategies.

Circulating molecular biomarkers—including microRNAs, long non-coding RNAs, extracellular vesicles, and proteomic signatures—offer potential for early diagnosis, patient stratification, and monitoring of treatment response. Conventional hormonal therapies, although partially effective in symptom management, do not adequately address the underlying systemic and molecular dysregulations. Innovative therapeutic strategies targeting angiogenesis, immune modulation, and epigenetic regulation, in combination with advanced delivery systems such as nanotherapeutics, provide promising avenues for precision medicine tailored to individual molecular endotypes.

Ultimately, a systems medicine framework affords a holistic paradigm to reconceptualize endometriosis as a systemic inflammatory disorder with gynecologic expression. Integrative multi-omics analyses, network-based modeling, and pathway-informed strategies enable a comprehensive understanding of disease mechanisms and guide the development of personalized therapeutic interventions. Advancing diagnostics, therapeutics, and patient care will require interdisciplinary collaboration across molecular biology, immunology, clinical practice, and bioinformatics. By moving beyond the lesion-centered paradigm, this perspective fosters a molecularly informed, patient-centered approach that addresses both local pathology and systemic consequences, with the potential to improve clinical outcomes and transform the management of endometriosis.

## Data Availability

No new data were created or analyzed in this study. Data sharing is not applicable to this article.

## Abbreviations

Akt	protein kinase B
BMP	bone morphogenetic protein
BCL6	B-cell lymphoma 6
CEACAM1	carcinoembryonic antigen-related cell adhesion molecule 1
COX-2	cyclooxygenase-2
CpG	cytosine-phosphate-guanine
CREB	cAMP response element-binding protein
cAMP	cyclic adenosine monophosphate
E1	estrone
E2	estradiol
E-cadherin	epithelial cadherin
EMT	epithelial–mesenchymal transition
eMSCs	endometrial mesenchymal stem cells
ER	estrogen receptor
ERs	estrogen receptors
EST	estrogen sulfotransferase
EV	extracellular vesicle
FBs	fibroblasts
FKBP52	FK506-binding protein 52
FLT1	fms-related tyrosine kinase 1
GLUT4	glucose transporter type 4
HIF-1	hypoxia-inducible factor 1
HIF-1α	hypoxia-inducible factor 1 alpha
HDACi	histone deacetylase inhibitors
HSD17β1	17β-hydroxysteroid dehydrogenase type 1
HSD17β2	17β-hydroxysteroid dehydrogenase type 2
IHH-COUPTFII-WNT4	Indian hedgehog–chicken ovalbumin upstream promoter transcription factor II–Wingless-related integration site 4 signaling cascade
IL	interleukin
IL-8	interleukin-8
JAK-STAT	Janus kinase–signal transducer and activator of transcription
MCP-1	monocyte chemoattractant protein-1
MAPK	mitogen-activated protein kinase
MMPs	matrix metalloproteinases
MSCs	mesenchymal stem cells
miR	microRNA
MRI	magnetic resonance imaging
NF-κB	nuclear factor kappa-light-chain-enhancer of activated B cells
NOTCH1	Notch receptor 1
N-cadherin	neural cadherin
PDH	pyruvate dehydrogenase
PDK1	pyruvate dehydrogenase kinase 1
PI3K	phosphoinositide 3-kinase
PIWIL2	Piwi-like RNA-mediated gene silencing 2
PGI2	prostacyclin
PGE2	prostaglandin E2
PKA	protein kinase A
PLCD1	phospholipase C delta 1
POLQ	DNA polymerase theta
PGR	progesterone receptor
Scn11A	sodium voltage-gated channel alpha subunit 11
SF-1	steroidogenic factor 1
Smad3	mothers against decapentaplegic homolog 3
Sp1/Sp3	specificity protein 1/specificity protein 3
StAR	steroidogenic acute regulatory protein
SIRT1	sirtuin 1
STS	steroid sulfatase
TEX41	testis-expressed 41
TGF-β	transforming growth factor beta
TGF-β1	transforming growth factor beta 1
TNF-α	tumor necrosis factor-alpha
WNT	Wingless-related integration site
VEGF	vascular endothelial growth factor
VEGFR2	vascular endothelial growth factor receptor 2

## References

[1] Czubak P., Herda K., Niewiadomska I., Putowski L., Łańcut M., Masłyk M. (2025). Understanding Endometriosis: A Broad Review of Its Causes, Management, and Impact. Int. J. Mol. Sci..

[2] As-Sanie S., Mackenzie S.C., Morrison L., Schrepf A., Zondervan K.T., Horne A.W., Missmer S.A. (2025). Endometriosis. JAMA.

[3] Soliman A.M., Surrey E., Bonafede M., Nelson J.K., Castelli-Haley J. (2018). Real-World Evaluation of Direct and Indirect Economic Burden Among Endometriosis Patients in the United States. Adv. Ther..

[4] Cousins F.L., McKinnon B.D., Mortlock S., Fitzgerald H.C., Zhang C., Montgomery G.W., Gargett C.E. (2023). New concepts on the etiology of endometriosis. J. Obstet. Gynaecol. Res..

[5] Horne A.W., Missmer S.A. (2022). Pathophysiology, diagnosis, and management of endometriosis. BMJ.

[6] Arafah M., Rashid S., Akhtar M. (2021). Endometriosis: A Comprehensive Review. Adv. Anat. Pathol..

[7] Bulun S., Monsivais D., Kakinuma T., Furukawa Y., Bernardi L., Pavone M., Dyson M., Bulun S.E. (2015). Molecular Biology of Endometriosis: From Aromatase to Genomic Abnormalities. Semin. Reprod. Med..

[8] Gupta S., Nguyen H.L., Morello F.A., Ahrar K., Wallace M.J., Madoff D.C., Murthy R., Hicks M.E. (2004). Various Approaches for CT-guided Percutaneous Biopsy of Deep Pelvic Lesions: Anatomic and Technical Considerations. RadioGraphics.

[9] Wei M., Meng S., Dai F., Xiao L., Mu X., Tang J., Liu Y. (2024). Comparison of two 3D reconstruction models for understanding of complicated female pelvic tumors. Int. J. Gynecol. Obstet..

[10] Song S., Wang J., Han J., Xuan Y., Zhi W., Wu Q. (2022). A case report of serpentine-like syndrome and review of literature. BMC Pregnancy Childbirth.

[11] Nougaret S., Nikolovski I., Paroder V., Vargas H.A., Sala E., Carrere S., Tetreau R., Hoeffel C., Forstner R., Lakhman Y. (2019). MRI of Tumors and Tumor Mimics in the Female Pelvis: Anatomic Pelvic Space–based Approach. RadioGraphics.

[12] Rousset P., Florin M., Bharwani N., Touboul C., Monroc M., Golfier F., Nougaret S., Thomassin-Naggara I. (2023). Deep pelvic infiltrating endometriosis: MRI consensus lexicon and compartment-based approach from the ENDOVALIRM group. Diagn. Interv. Imaging.

[13] Garcia Garcia J.M., Vannuzzi V., Donati C., Bernacchioni C., Bruni P., Petraglia F. (2023). Endometriosis: Cellular and Molecular Mechanisms Leading to Fibrosis. Reprod. Sci..

[14] Marino Y., Inferrera F., Genovese T., Cuzzocrea S., Fusco R., Di Paola R. (2025). Mitochondrial dynamics: Molecular mechanism and implications in endometriosis. Biochimie.

[15] Pszczołowska M., Walczak K., Kołodziejczyk W., Kozłowska M., Kozłowski G., Gachowska M., Leszek J. (2025). Understanding Deep Endometriosis: From Molecular to Neuropsychiatry Dimension. Int. J. Mol. Sci..

[16] Mariadas H., Chen J.-H., Chen K.-H. (2025). The Molecular and Cellular Mechanisms of Endometriosis: From Basic Pathophysiology to Clinical Implications. Int. J. Mol. Sci..

[17] Smolarz B., Szyłło K., Romanowicz H. (2021). Endometriosis: Epidemiology, Classification, Pathogenesis, Treatment and Genetics (Review of Literature). Int. J. Mol. Sci..

[18] Pašalić E., Tambuwala M.M., Hromić-Jahjefendić A. (2023). Endometriosis: Classification, pathophysiology, and treatment options. Pathol. Res. Pract..

[19] Mahdavi R., Akbari Jonoush Z., Mohammadi N., Mousavi Salehi A., Ghadiri N., Sayadi M., Behravan M., Moramezi F., Ghafourian M., Farzaneh M. (2025). Dysregulation of Key Biological Processes in Endometriosis Pathophysiology. Curr. Mol. Med..

[20] Gunther K., Fisher T., Liu D., Abbott J., Ford C.E. (2025). Endometriosis is not the endometrium: Reviewing the over-representation of eutopic endometrium in endometriosis research. Elife.

[21] Bedrick B.S., Courtright L., Zhang J., Snow M., Sampaio Amendola I.L., Nylander E., Cayton-Vaught K., Segars J., Singh B. (2024). A systematic review of epigenetics of endometriosis. F S Rev..

[22] Poli-Neto O.B., Meola J., Rosa-e-Silva J.C., Tiezzi D. (2020). Transcriptome meta-analysis reveals differences of immune profile between eutopic endometrium from stage I-II and III-IV endometriosis independently of hormonal milieu. Sci. Rep..

[23] Feng X., Qi L., Xu X., Feng Y., Gong X., Aili A., Chen Y., Xue Z., Xue J., Tong X. (2021). Analysis of differences in the transcriptomic profiles of eutopic and ectopic endometriums in women with ovarian endometriosis. PeerJ.

[24] Zheng J., Dai Y., Lin X., Huang Q., Shi L., Jin X., Liu N., Zhou F., Zhang S. (2021). Hypoxia-induced lactate dehydrogenase A protects cells from apoptosis in endometriosis. Mol. Med. Rep..

[25] Sarsenova M., Lawarde A., Pathare A.D.S., Saare M., Modhukur V., Soplepmann P., Terasmaa A., Käämbre T., Gemzell-Danielsson K., Lalitkumar P.G.L. (2024). Endometriotic lesions exhibit distinct metabolic signature compared to paired eutopic endometrium at the single-cell level. Commun. Biol..

[26] Liu S., Li X., Gu Z., Wu J., Jia S., Shi J., Dai Y., Wu Y., Yan H., Zhang J. (2025). Single-cell and spatial transcriptomic profiling revealed niche interactions sustaining growth of endometriotic lesions. Cell Genom..

[27] Ma J., Zhang L., Zhan H., Mo Y., Ren Z., Shao A., Lin J. (2021). Single-cell transcriptomic analysis of endometriosis provides insights into fibroblast fates and immune cell heterogeneity. Cell Biosci..

[28] Marquardt R.M., Tran D.N., Lessey B.A., Rahman M.S., Jeong J.-W. (2023). Epigenetic Dysregulation in Endometriosis: Implications for Pathophysiology and Therapeutics. Endocr. Rev..

[29] MacLean J.A., Hayashi K. (2022). Progesterone Actions and Resistance in Gynecological Disorders. Cells.

[30] Zhang H., Sheng S., Pan Z., Zhao L., Yang C., Li C., Wang F. (2023). Immune and endocrine regulation in endometriosis: What we know. J. Endometr. Uterine Disord..

[31] Greygoose E., Metharom P., Kula H., Seckin T.K., Seckin T.A., Ayhan A., Yu Y. (2025). The Estrogen–Immune Interface in Endometriosis. Cells.

[32] Chantalat E., Valera M.-C., Vaysse C., Noirrit E., Rusidze M., Weyl A., Vergriete K., Buscail E., Lluel P., Fontaine C. (2020). Estrogen Receptors and Endometriosis. Int. J. Mol. Sci..

[33] Mori T., Ito F., Koshiba A., Kataoka H., Takaoka O., Okimura H., Khan K.N., Kitawaki J. (2019). Local estrogen formation and its regulation in endometriosis. Reprod. Med. Biol..

[34] Qi Q.-M., Guo S.-W., Liu X.-S. (2017). Estrogen Biosynthesis and Its Regulation in Endometriosis. Reprod. Dev. Med..

[35] Zhang P., Wang G. (2023). Progesterone Resistance in Endometriosis: Current Evidence and Putative Mechanisms. Int. J. Mol. Sci..

[36] Rocha C.V., Da Broi M.G., Miranda-Furtado C.L., Navarro P.A., Ferriani R.A., Meola J. (2019). Progesterone Receptor B (PGR-B) Is Partially Methylated in Eutopic Endometrium From Infertile Women With Endometriosis. Reprod. Sci..

[37] Bulun S.E., Gurates B., Fang Z., Tamura M., Sebastian S., Zhou J., Amin S., Yang S. (2002). Mechanisms of excessive estrogen formation in endometriosis. J. Reprod. Immunol..

[38] García-Gómez E., Vázquez-Martínez E.R., Reyes-Mayoral C., Cruz-Orozco O.P., Camacho-Arroyo I., Cerbón M. (2020). Regulation of Inflammation Pathways and Inflammasome by Sex Steroid Hormones in Endometriosis. Front. Endocrinol..

[39] Dai W., Guo R., Na X., Jiang S., Liang J., Guo C., Fang Y., Na Z., Li D. (2024). Hypoxia and the endometrium: An indispensable role for HIF-1α as therapeutic strategies. Redox Biol..

[40] Zhang T., Wang Y., Wang Y., Liu C., Han C. (2022). Crosstalk between Extracellular Matrix Stiffness and ROS Drives Endometrial Repair via the HIF-1α/YAP Axis during Menstruation. Cells.

[41] Wang S., Chen X., Guo S., Zhou F., Zhang X., Lu C., Yang X., Wang Q., He B., Wang J. (2023). CXCR4, regulated by HIF1A, promotes endometrial breakdown via CD45+ leukocyte recruitment in a mouse model of menstruation. Reprod. Biol..

[42] Zhang L., Xiong W., Fu T., Long X., Zhang Z., Liu Y., Lv G. (2020). Oestrogen receptors and hypoxia inducible factor 1 alpha expression in abdominal wall endometriosis. Reprod. Biomed. Online.

[43] Dai Y., Lin X., Xu W., Lin X., Huang Q., Shi L., Pan Y., Zhang Y., Zhu Y., Li C. (2019). MiR-210-3p protects endometriotic cells from oxidative stress-induced cell cycle arrest by targeting BARD1. Cell Death Dis..

[44] Zhang L., Liu H., Xiong W., He H., Fu T., Long X., Li X., Liang J., Ding H., Xu Y. (2024). *CircFOXO3* mediates hypoxia-induced autophagy of endometrial stromal cells in endometriosis. FASEB J..

[45] Lin X., Dai Y., Xu W., Shi L., Jin X., Li C., Zhou F., Pan Y., Zhang Y., Lin X. (2018). Hypoxia Promotes Ectopic Adhesion Ability of Endometrial Stromal Cells via TGF-β1/Smad Signaling in Endometriosis. Endocrinology.

[46] Peng H., Weng L., Lei S., Hou S., Yang S., Li M., Zhao D. (2022). Hypoxia-hindered methylation of PTGIS in endometrial stromal cells accelerates endometriosis progression by inducing CD16− NK-cell differentiation. Exp. Mol. Med..

[47] Dudley A.C., Griffioen A.W. (2023). Pathological angiogenesis: Mechanisms and therapeutic strategies. Angiogenesis.

[48] Saunders P.T.K., Horne A.W. (2021). Endometriosis: Etiology, pathobiology, and therapeutic prospects. Cell.

[49] Li W.-N., Wu M.-H., Tsai S.-J. (2021). HYPOXIA AND REPRODUCTIVE HEALTH: The role of hypoxia in the development and progression of endometriosis. Reproduction.

[50] Samimi M., Pourhanifeh M.H., Mehdizadehkashi A., Eftekhar T., Asemi Z. (2019). The role of inflammation, oxidative stress, angiogenesis, and apoptosis in the pathophysiology of endometriosis: Basic science and new insights based on gene expression. J. Cell Physiol..

[51] Nati I.D., Malutan A., Ciortea R., Oancea M., Bucuri C., Roman M., Ormindean C., Milon A.G., Mihu D. (2024). Exploring the Influence of IL-8, IL-10, Patient-Reported Pain, and Physical Activity on Endometriosis Severity. Diagnostics.

[52] Nishimoto-Kakiuchi A., Sato I., Nakano K., Ohmori H., Kayukawa Y., Tanimura H., Yamamoto S., Sakamoto Y., Nakamura G., Maeda A. (2023). A long-acting anti–IL-8 antibody improves inflammation and fibrosis in endometriosis. Sci. Transl. Med..

[53] Rahmawati N.Y., Ahsan F., Santoso B., Mufid A.F., Sa’adi A., Dwiningsih S.R., Tunjungseto A., Widyanugraha M.Y.A. (2023). IL-8 and IL-12p70 are associated with pelvic pain among infertile women with endometriosis. Pain Med..

[54] Oală I.E., Mitranovici M.-I., Chiorean D.M., Irimia T., Crișan A.I., Melinte I.M., Cotruș T., Tudorache V., Moraru L., Moraru R. (2024). Endometriosis and the Role of Pro-Inflammatory and Anti-Inflammatory Cytokines in Pathophysiology: A Narrative Review of the Literature. Diagnostics.

[55] Zhou W.-J., Yang H.-L., Shao J., Mei J., Chang K.-K., Zhu R., Li M.-Q. (2019). Anti-inflammatory cytokines in endometriosis. Cell. Mol. Life Sci..

[56] Yi Y., Nie J., Liu X., Guo S.-W. (2025). Progressively Diminished Prostaglandin E2 Signaling in Concordance with Increasing Fibrosis in Ectopic Endometrium. Reprod. Sci..

[57] Kodarahmian M., Amidi F., Moini A., Kashani L., Shabani Nashtaei M., Pazhohan A., Bahramrezai M., Berenjian S., Sobhani A. (2019). The modulating effects of Resveratrol on the expression of MMP-2 and MMP-9 in endometriosis women: A randomized exploratory trial. Gynecol. Endocrinol..

[58] Huang Q., Song Y., Lei X., Huang H., Nong W. (2024). MMP-9 as a clinical marker for endometriosis: A meta-analysis and bioinformatics analysis. Front. Endocrinol..

[59] Augoulea A., Kindis A., Karopoulou E., Tsoltos N., Kaparos G., Tsakonas E., Panoulis K. (2020). Age at Menarche and Oxidative Stress Markers in Women with Endometriosis. SN Compr. Clin. Med..

[60] Baradwan S., Gari A., Sabban H., Alshahrani M.S., Khadawardi K., Bukhari I.A., Alyousef A., Abu-Zaid A. (2024). The effect of antioxidant supplementation on dysmenorrhea and endometriosis-associated painful symptoms: A systematic review and meta-analysis of randomized clinical trials. Obstet. Gynecol. Sci..

[61] Ke J., Ye J., Li M., Zhu Z. (2021). The Role of Matrix Metalloproteinases in Endometriosis: A Potential Target. Biomolecules.

[62] D’Amico G., Muñoz-Félix J.M., Pedrosa A.R., Hodivala-Dilke K.M. (2020). “Splitting the matrix”: Intussusceptive angiogenesis meets MT1-MMP. EMBO Mol. Med..

[63] Apte R.S., Chen D.S., Ferrara N. (2019). VEGF in Signaling and Disease: Beyond Discovery and Development. Cell.

[64] Guo F., He Y., Fan Y., Du Z., Sun H., Feng Z., Zhang G., Xiong T. (2021). G-CSF and IL-6 may be involved in formation of endometriosis lesions by increasing the expression of angiogenic factors in neutrophils. Mol. Hum. Reprod..

[65] Chen S., Liu Y., Zhong Z., Wei C., Liu Y., Zhu X. (2023). Peritoneal immune microenvironment of endometriosis: Role and therapeutic perspectives. Front. Immunol..

[66] Vallvé-Juanico J., Houshdaran S., Giudice L.C. (2019). The endometrial immune environment of women with endometriosis. Hum. Reprod. Update.

[67] Shigesi N., Kvaskoff M., Kirtley S., Feng Q., Fang H., Knight J.C., Missmer S.A., Rahmioglu N., Zondervan K.T., Becker C.M. (2019). The association between endometriosis and autoimmune diseases: A systematic review and meta-analysis. Hum. Reprod. Update.

[68] Shafrir A.L., Palmor M.C., Fourquet J., DiVasta A.D., Farland L.V., Vitonis A.F., Harris H.R., Laufer M.R., Cramer D.W., Terry K.L. (2021). Co-occurrence of immune-mediated conditions and endometriosis among adolescents and adult women. Am. J. Reprod. Immunol..

[69] Blanco L.P., Salmeri N., Temkin S.M., Shanmugam V.K., Stratton P. (2025). Endometriosis and autoimmunity. Autoimmun. Rev..

[70] Yamada Y., Uchiyama T., Ito F., Kawahara N., Ogawa K., Obayashi C., Kobayashi H. (2019). Clinical significance of M2 macrophages expressing heme oxygenase-1 in malignant transformation of ovarian endometrioma. Pathol. Res. Pract..

[71] Ramírez-Pavez T.N., Martínez-Esparza M., Ruiz-Alcaraz A.J., Marín-Sánchez P., Machado-Linde F., García-Peñarrubia P. (2021). The Role of Peritoneal Macrophages in Endometriosis. Int. J. Mol. Sci..

[72] Meggyes M., Szereday L., Bohonyi N., Koppan M., Szegedi S., Marics-Kutas A., Marton M., Totsimon A., Polgar B. (2020). Different Expression Pattern of TIM-3 and Galectin-9 Molecules by Peripheral and Peritoneal Lymphocytes in Women with and without Endometriosis. Int. J. Mol. Sci..

[73] Hanada T., Tsuji S., Nakayama M., Wakinoue S., Kasahara K., Kimura F., Mori T., Ogasawara K., Murakami T. (2018). Suppressive regulatory T cells and latent transforming growth factor-β-expressing macrophages are altered in the peritoneal fluid of patients with endometriosis. Reprod. Biol. Endocrinol..

[74] Riccio L.G.C., Baracat E.C., Chapron C., Batteux F., Abrão M.S. (2017). The role of the B lymphocytes in endometriosis: A systematic review. J. Reprod. Immunol..

[75] Dai Y., Ye Z., Lin X., Zhang S. (2025). Immunopathological insights into endometriosis: From research advances to future treatments. Semin. Immunopathol..

[76] Guo M., Bafligil C., Tapmeier T., Hubbard C., Manek S., Shang C., Martinez F.O., Schmidt N., Obendorf M., Hess-Stumpp H. (2020). Mass cytometry analysis reveals a distinct immune environment in peritoneal fluid in endometriosis: A characterisation study. BMC Med..

[77] Huang Z., Lin D., Zhang H., Yang M., Chen J., Ding X., Dai S., Hong Y., Liang G., Li Q. (2024). The dysfunction of CD8^+^ T cells triggered by endometriotic stromal cells promotes the immune survival of endometriosis. Immunology.

[78] Hoogstad-van Evert J., Paap R., Nap A., van der Molen R. (2022). The Promises of Natural Killer Cell Therapy in Endometriosis. Int. J. Mol. Sci..

[79] Ono Y., Yoshino O., Hiraoka T., Sato E., Furue A., Nawaz A., Hatta H., Fukushi Y., Wada S., Tobe K. (2021). CD206+ macrophage is an accelerator of endometriotic-like lesion via promoting angiogenesis in the endometriosis mouse model. Sci. Rep..

[80] Ahmed R.S., Sherif M., Alghamdi M.A., El-Tallawy S.N., Alzaydan O.K., Pergolizzi J.V., Varrassi G., Zaghra Z., Abdelsalam Z.S., Kamal M.T. (2025). Exploring the Immune System’s Role in Endometriosis: Insights Into Pathogenesis, Pain, and Treatment. Cureus.

[81] Olkowska-Truchanowicz J., Sztokfisz-Ignasiak A., Zwierzchowska A., Janiuk I., Dąbrowski F., Korczak-Kowalska G., Barcz E., Bocian K., Malejczyk J. (2021). Endometriotic Peritoneal Fluid Stimulates Recruitment of CD4+CD25highFOXP3+ Treg Cells. J. Clin. Med..

[82] Hou X.-X., Wang X.-Q., Zhou W.-J., Li D.-J. (2021). Regulatory T cells induce polarization of pro-repair macrophages by secreting sFGL2 into the endometriotic milieu. Commun. Biol..

[83] Izumi G., Koga K., Takamura M., Makabe T., Nagai M., Urata Y., Harada M., Hirata T., Hirota Y., Fujii T. (2017). Mannose receptor is highly expressed by peritoneal dendritic cells in endometriosis. Fertil. Steril..

[84] Qiaomei Z., Ping W., Yanjing Z., Jinhua W., Shaozhan C., Lihong C. (2023). Features of peritoneal dendritic cells in the development of endometriosis. Reprod. Biol. Endocrinol..

[85] Suen J., Chang Y., Shiu Y., Hsu C., Sharma P., Chiu C., Chen Y., Hour T., Tsai E. (2019). IL-10 from plasmacytoid dendritic cells promotes angiogenesis in the early stage of endometriosis. J. Pathol..

[86] Soroczynska K., Zareba L., Dlugolecka M., Czystowska-Kuzmicz M. (2022). Immunosuppressive Extracellular Vesicles as a Linking Factor in the Development of Tumor and Endometriotic Lesions in the Gynecologic Tract. Cells.

[87] Kusunoki M., Fujiwara Y., Komohara Y., Imamura Y., Honda R., Ohba T., Katabuchi H. (2021). Hemoglobin-induced continuous activation of macrophages in endometriotic cysts: A potential mechanism of endometriosis development and carcinogenesis. Med. Mol. Morphol..

[88] Crispim P.C.A., Jammal M.P., Antão P.K.A., Micheli D.C., Tavares-Murta B.M., Murta E.F.C., Nomelini R.S. (2020). IL6, IL8, and IL10 in the distinction of malignant ovarian neoplasms and endometriomas. Am. J. Reprod. Immunol..

[89] AlAshqar A., Reschke L., Kirschen G.W., Borahay M.A. (2021). Role of inflammation in benign gynecologic disorders: From pathogenesis to novel therapies. Biol. Reprod..

[90] Li Y., Li R., Ouyang N., Dai K., Yuan P., Zheng L., Wang W. (2019). Investigating the impact of local inflammation on granulosa cells and follicular development in women with ovarian endometriosis. Fertil. Steril..

[91] Ghodsi M., Hojati V., Attaranzadeh A., Saifi B. (2022). Evaluation of IL-3, IL-5, and IL-6 concentration in the follicular fluid of women with endometriosis: A cross-sectional study. Int. J. Reprod. Biomed..

[92] Wang X., Wei Z., Tang Z., Xue C., Yu H., Zhang D., Li Y., Liu X., Shi Y., Zhang L. (2021). IL-37bΔ1-45 suppresses the migration and invasion of endometrial cancer cells by targeting the Rac1/NF-κB/MMP2 signal pathway. Lab. Investig..

[93] Ganieva U., Nakamura T., Osuka S., Bayasula Nakanishi N., Kasahara Y., Takasaki N., Muraoka A., Hayashi S., Nagai T., Murase T. (2020). Involvement of Transcription Factor 21 in the Pathogenesis of Fibrosis in Endometriosis. Am. J. Pathol..

[94] Knific T., Fishman D., Vogler A., Gstöttner M., Wenzl R., Peterson H., Rižner T.L. (2019). Multiplex analysis of 40 cytokines do not allow separation between endometriosis patients and controls. Sci. Rep..

[95] Zarezadeh Mehrabadi A., Aghamohamadi N., Khoshmirsafa M., Aghamajidi A., Pilehforoshha M., Massoumi R., Falak R. (2022). The roles of interleukin-1 receptor accessory protein in certain inflammatory conditions. Immunology.

[96] Huang J., Chen X., Lv Y. (2021). HMGB1 Mediated Inflammation and Autophagy Contribute to Endometriosis. Front. Endocrinol..

[97] Zasheva D., Dimitrov R., Stamenova M. (2007). Endometriosis and the role of the integrins in the pathogenesis of the endometriosis. Akush Ginekol..

[98] Sillem M., Prifti S., Monga B., Arslic T., Runnebaum B. (1999). Integrin-mediated adhesion of uterine endometrial cells from endometriosis patients to extracellular matrix proteins is enhanced by tumor necrosis factor alpha (TNFα) and interleukin-1 (IL-1). Eur. J. Obstet. Gynecol. Reprod. Biol..

[99] Zhang L., Li H., Yuan M., Li D., Sun C., Wang G. (2020). Serum Exosomal MicroRNAs as Potential Circulating Biomarkers for Endometriosis. Dis. Markers.

[100] Huang Y., Zhu L., Li H., Ye J., Lin N., Chen M., Pan D., Chen Z. (2022). Endometriosis derived exosomal miR-301a-3p mediates macrophage polarization via regulating PTEN-PI3K axis. Biomed. Pharmacother..

[101] Allard B., Longhi M.S., Robson S.C., Stagg J. (2017). The ectonucleotidases CD 39 and CD 73: Novel checkpoint inhibitor targets. Immunol. Rev..

[102] Shao J., Zhang B., Yu J.-J., Wei C.-Y., Zhou W.-J., Chang K.-K., Yang H.-L., Jin L.-P., Zhu X.-Y., Li M.-Q. (2016). Macrophages promote the growth and invasion of endometrial stromal cells by downregulating IL-24 in endometriosis. Reproduction.

[103] Liu T., Liu M., Zheng C., Zhang D., Li M., Zhang L. (2021). Exosomal lncRNA CHL1-AS1 Derived from Peritoneal Macrophages Promotes the Progression of Endometriosis via the miR-610/MDM2 Axis. Int. J. Nanomed..

[104] Sun H., Li D., Yuan M., Li Q., Zhen Q., Li N., Wang G. (2019). Macrophages alternatively activated by endometriosis-exosomes contribute to the development of lesions in mice. MHR Basic Sci. Reprod. Med..

[105] Schjenken J.E., Panir K., Robertson S.A., Hull M.L. (2019). Exosome-mediated intracellular signalling impacts the development of endometriosis—New avenues for endometriosis research. MHR Basic Sci. Reprod. Med..

[106] Santoso B., Sa’adi A., Dwiningsih S.R., Tunjungseto A., Widyanugraha M.Y.A., Mufid A.F., Rahmawati N.Y., Ahsan F. (2020). Soluble immune checkpoints CTLA-4, HLA-G, PD-1, and PD-L1 are associated with endometriosis-related infertility. Am. J. Reprod. Immunol..

[107] Yao H., Wang H., Li C., Fang J.-Y., Xu J. (2018). Cancer Cell-Intrinsic PD-1 and Implications in Combinatorial Immunotherapy. Front. Immunol..

[108] Suszczyk D., Skiba W., Zardzewiały W., Pawłowska A., Włodarczyk K., Polak G., Tarkowski R., Wertel I. (2022). Clinical Value of the PD-1/PD-L1/PD-L2 Pathway in Patients Suffering from Endometriosis. Int. J. Mol. Sci..

[109] Hosseinzadeh R., Moini A., Hosseini R., Fatehnejad M., Yekaninejad M.S., Javidan M., Changaei M., Feizisani F., Rajaei S. (2024). A higher number of exhausted local PD1+, but not TIM3+, NK cells in advanced endometriosis. Heliyon.

[110] Emond J.-P., Caron P., Pušić M., Turcotte V., Simonyan D., Vogler A., Osredkar J., Rižner T.L., Guillemette C. (2023). Circulating estradiol and its biologically active metabolites in endometriosis and in relation to pain symptoms. Front. Endocrinol..

[111] Murakami K., Kotani Y., Nakai H., Matsumura N. (2020). Endometriosis-Associated Ovarian Cancer: The Origin and Targeted Therapy. Cancers.

[112] Suszczyk D., Skiba W., Pawłowska-Łachut A., Dymanowska-Dyjak I., Włodarczyk K., Paduch R., Wertel I. (2024). Immune Checkpoints in Endometriosis—A New Insight in the Pathogenesis. Int. J. Mol. Sci..

[113] Zhang Y., Zhang H., Yan L., Liang G., Zhu C., Wang Y., Ji S., He C., Sun J., Zhang J. (2023). Exosomal microRNAs in tubal fluid may be involved in damage to tubal reproductive function associated with tubal endometriosis. Reprod. Biomed. Online.

[114] Bjorkman S., Taylor H.S. (2019). MicroRNAs in endometriosis: Biological function and emerging biomarker candidates. Biol. Reprod..

[115] Panir K., Schjenken J.E., Robertson S.A., Hull M.L. (2018). Non-coding RNAs in endometriosis: A narrative review. Hum. Reprod. Update.

[116] Zhang A., Wang G., Jia L., Su T., Zhang L. (2018). Exosome-mediated microRNA-138 and vascular endothelial growth factor in endometriosis through inflammation and apoptosis via the nuclear factor-κB signaling pathway. Int. J. Mol. Med..

[117] Zhang Z., Li H., Zhao Z., Gao B., Meng L., Feng X. (2019). miR-146b level and variants is associated with endometriosis related macrophages phenotype and plays a pivotal role in the endometriotic pain symptom. Taiwan. J. Obstet. Gynecol..

[118] Wu M., Zhang Y. (2021). MiR-182 inhibits proliferation, migration, invasion and inflammation of endometrial stromal cells through deactivation of NF-κB signaling pathway in endometriosis. Mol. Cell Biochem..

[119] Nematian S.E., Mamillapalli R., Kadakia T.S., Majidi Zolbin M., Moustafa S., Taylor H.S. (2018). Systemic Inflammation Induced by microRNAs: Endometriosis-Derived Alterations in Circulating microRNA 125b-5p and Let-7b-5p Regulate Macrophage Cytokine Production. J. Clin. Endocrinol. Metab..

[120] Pei T., Liu C., Liu T., Xiao L., Luo B., Tan J., Li X., Zhou G., Duan C., Huang W. (2018). miR-194-3p Represses the Progesterone Receptor and Decidualization in Eutopic Endometrium From Women With Endometriosis. Endocrinology.

[121] Lin S.-C., Li W.-N., Lin S.-C., Hou H.-T., Tsai Y.-C., Lin T.-C., Wu M.-H., Tsai S.-J. (2023). Targeting YAP1 ameliorates progesterone resistance in endometriosis. Hum. Reprod..

[122] Papari E., Noruzinia M., Kashani L., Foster W.G. (2020). Identification of candidate microRNA markers of endometriosis with the use of next-generation sequencing and quantitative real-time polymerase chain reaction. Fertil. Steril..

[123] Nothnick W.B. (2022). MicroRNAs and Progesterone Receptor Signaling in Endometriosis Pathophysiology. Cells.

[124] Meng X., Liu J., Wang H., Chen P., Wang D. (2019). MicroRNA-126-5p downregulates BCAR3 expression to promote cell migration and invasion in endometriosis. Mol. Cell Endocrinol..

[125] Li N., Yi K., Li X., Wang Y., Jing J., Hu J., Wang Z. (2022). MiR-143-3p facilitates motility and invasiveness of endometriotic stromal cells by targeting VASH1/TGF-β signaling. Reprod. Biol..

[126] Goetz T.G., Mamillapalli R., Taylor H.S. (2016). Low Body Mass Index in Endometriosis Is Promoted by Hepatic Metabolic Gene Dysregulation in Mice1. Biol Reprod..

[127] Horton J., Sterrenburg M., Lane S., Maheshwari A., Li T.C., Cheong Y. (2019). Reproductive, obstetric, and perinatal outcomes of women with adenomyosis and endometriosis: A systematic review and meta-analysis. Hum. Reprod. Update.

[128] Surrey E.S., Soliman A.M., Agarwal S.K., Snabes M.C., Diamond M.P. (2019). Impact of elagolix treatment on fatigue experienced by women with moderate to severe pain associated with endometriosis. Fertil. Steril..

[129] Gao M., Koupil I., Sjöqvist H., Karlsson H., Lalitkumar S., Dalman C., Kosidou K. (2020). Psychiatric comorbidity among women with endometriosis: Nationwide cohort study in Sweden. Am. J. Obstet. Gynecol..

[130] Okoth K., Wang J., Zemedikun D., Thomas G., Nirantharakumar K., Adderley N. (2021). Risk of cardiovascular outcomes among women with endometriosis in the United Kingdom: A retrospective matched cohort study. BJOG Int. J. Obstet. Gynaecol..

[131] Moustafa S., Burn M., Mamillapalli R., Nematian S., Flores V., Taylor H.S. (2020). Accurate diagnosis of endometriosis using serum microRNAs. Am. J. Obstet. Gynecol..

[132] Taylor H.S. (2021). Reimagining Endometriosis. Med.

[133] Li T., Mamillapalli R., Ding S., Chang H., Liu Z.-W., Gao X.-B., Taylor H.S. (2018). Endometriosis alters brain electrophysiology, gene expression and increases pain sensitization, anxiety, and depression in female mice. Biol. Reprod..

[134] Clower L., Fleshman T., Geldenhuys W.J., Santanam N. (2022). Targeting Oxidative Stress Involved in Endometriosis and Its Pain. Biomolecules.

[135] Tamura K., Yoshie M., Kusama K., Tsuru A. (2025). Mechanisms of Decidual Dysfunction and Infertility in Endometriosis: Roles of Prostaglandins and SASP. Reprod. Med. Biol..

[136] Kusama K., Satoyoshi A., Azumi M., Yoshie M., Kojima J., Mizuno Y., Ono M., Nishi H., Kajihara T., Tamura K. (2022). Toll-like receptor signaling pathway triggered by inhibition of serpin A1 stimulates production of inflammatory cytokines by endometrial stromal cells. Front. Endocrinol..

[137] Bedaiwy M.A. (2002). Prediction of endometriosis with serum and peritoneal fluid markers: A prospective controlled trial. Hum. Reprod..

[138] Akoum A., Al-Akoum M., Lemay A., Maheux R., Leboeuf M. (2008). Imbalance in the peritoneal levels of interleukin 1 and its decoy inhibitory receptor type II in endometriosis women with infertility and pelvic pain. Fertil. Steril..

[139] Ho H., Wu M., Yang Y. (1997). Peritoneal Cellular Immunity and Endometriosis. Am. J. Reprod. Immunol..

[140] Koumantakis E., Matalliotakis I., Neonaki M., Froudarakis G., Georgoulias V. (1994). Soluble serum interleukin-2 receptor, interleukin-6 and interleukin-1a in patients with endometriosis and in controls. Arch. Gynecol. Obstet..

[141] Mu F., Harris H.R., Rich-Edwards J.W., Hankinson S.E., Rimm E.B., Spiegelman D., Missmer S.A. (2018). A Prospective Study of Inflammatory Markers and Risk of Endometriosis. Am. J. Epidemiol..

[142] Martínez S., Garrido N., Coperias J.L., Pardo F., Desco J., García-Velasco J.A., Simón C., Pellicer A. (2007). Serum interleukin-6 levels are elevated in women with minimal–mild endometriosis. Hum. Reprod..

[143] Xavier P., Belo L., Beires J., Rebelo I., Martinez-de-Oliveira J., Lunet N., Barros H. (2006). Serum levels of VEGF and TNF-α and their association with C-reactive protein in patients with endometriosis. Arch. Gynecol. Obstet..

[144] Pizzo A., Salmeri F.M., Ardita F.V., Sofo V., Tripepi M., Marsico S. (2002). Behaviour of Cytokine Levels in Serum and Peritoneal Fluid of Women with Endometriosis. Gynecol. Obstet. Investig..

[145] Viganò P., Parazzini F., Somigliana E., Vercellini P. (2004). Endometriosis: Epidemiology and aetiological factors. Best Pract. Res. Clin. Obstet. Gynaecol..

[146] Kany S., Vollrath J.T., Relja B. (2019). Cytokines in Inflammatory Disease. Int. J. Mol. Sci..

[147] Taylor H.S., Kotlyar A.M., Flores V.A. (2021). Endometriosis is a chronic systemic disease: Clinical challenges and novel innovations. Lancet.

[148] Benaglia L., Giacomini E., Reschini M., Pavone V., Ottolina J., Pagliardini L., Villanacci R., Papaleo E., La Vecchia I., Somigliana E. (2024). EXAMINING miRNA PROFILES IN PERIPHERAL BLOOD EXTRACELLULAR VESICLES: A COMPARISON BETWEEN PATIENTS WITH AND WITHOUT ENDOMETRIOSIS. Fertil. Steril..

[149] Machairiotis N., Vasilakaki S., Thomakos N. (2021). Inflammatory Mediators and Pain in Endometriosis: A Systematic Review. Biomedicines.

[150] Bashir S.T., Redden C.R., Raj K., Arcanjo R.B., Stasiak S., Li Q., Steelman A.J., Nowak R.A. (2023). Endometriosis leads to central nervous system-wide glial activation in a mouse model of endometriosis. J. Neuroinflamm..

[151] Barcelon E.E., Cho W.-H., Jun S.B., Lee S.J. (2019). Brain Microglial Activation in Chronic Pain-Associated Affective Disorder. Front. Neurosci..

[152] Šutulović N., Grubač Ž., Šuvakov S., Jovanović Đ., Puškaš N., Macut Đ., Marković A.R., Simić T., Stanojlović O., Hrnčić D. (2019). Chronic prostatitis/chronic pelvic pain syndrome increases susceptibility to seizures in rats and alters brain levels of IL-1β and IL-6. Epilepsy Res..

[153] Lopes F., Vicentini F.A., Cluny N.L., Mathews A.J., Lee B.H., Almishri W.A., Griffin L., Gonçalves W., Pinho V., McKay D.M. (2020). Brain TNF drives post-inflammation depression-like behavior and persistent pain in experimental arthritis. Brain Behav. Immun..

[154] Liu L.-L., Li J.-M., Su W.-J., Wang B., Jiang C.-L. (2019). Sex differences in depressive-like behaviour may relate to imbalance of microglia activation in the hippocampus. Brain Behav. Immun..

[155] Bekhbat M., Neigh G.N. (2018). Sex differences in the neuro-immune consequences of stress: Focus on depression and anxiety. Brain Behav. Immun..

[156] Yang F.-C., Zhou Y., Zhang S.-Y., Ma R. (2025). Depression and anxiety in patients with endometriosis-associated chronic pain: Neuroimmune mechanisms mediated by inflammatory factors. World J. Psychiatry.

[157] Wang Y., Li B., Zhou Y., Wang Y., Han X., Zhang S., He Z., Ouyang L. (2021). Does Endometriosis Disturb Mental Health and Quality of Life? A Systematic Review and Meta-Analysis. Gynecol. Obstet. Investig..

[158] Vöckel J., Markser A., Wege L., Wunram H.L., Sigrist C., Koenig J. (2024). Pharmacological anti-inflammatory treatment in children and adolescents with depressive symptoms: A systematic-review and meta-analysis. Eur. Neuropsychopharmacol..

[159] Miller A.H., Raison C.L. (2016). The role of inflammation in depression: From evolutionary imperative to modern treatment target. Nat. Rev. Immunol..

[160] Vardaman M., Gilmour S. (2025). Letter: Time to correct the record on the global burden of myalgic encephalomyelitis/chronic fatigue syndrome (ME/CFS). J. Transl. Med..

[161] Mokhtari T., Irandoost E., Sheikhbahaei F. (2024). Stress, pain, anxiety, and depression in endometriosis–Targeting glial activation and inflammation. Int. Immunopharmacol..

[162] Xu X., Mei J., Zhang B., Jiang X., Wang L., Zhang A., Li J., Chen S., He Y., Fang Y. (2025). Association Between Circulating Cytokines and Endometriosis: A Mendelian Randomization Study. J. Cell Mol. Med..

[163] Ping Z., Wen Z., Jinhua L., Jinghe L. (2019). Research on central sensitization of endometriosis-associated pain: A systematic review of the literature. J. Pain Res..

[164] Wirth K.J., Löhn M. (2023). Myalgic Encephalomyelitis/Chronic Fatigue Syndrome (ME/CFS) and Comorbidities: Linked by Vascular Pathomechanisms and Vasoactive Mediators?. Medicina.

[165] Holmer B.J., Lapierre S.S., Jake-Schoffman D.E., Christou D.D. (2021). Effects of sleep deprivation on endothelial function in adult humans: A systematic review. Geroscience.

[166] Compton S., Alkabalan R., Cadet J., Mastali A., Ramdass P.V.A.K. (2025). Endometriosis and Myalgic Encephalomyelitis/Chronic Fatigue Syndrome: A Systematic Review and Meta-Analysis. Diagnostics.

[167] Koninckx P.R., Ussia A., Adamyan L., Tahlak M., Keckstein J., Wattiez A., Martin D.C. (2021). The epidemiology of endometriosis is poorly known as the pathophysiology and diagnosis are unclear. Best Pract. Res. Clin. Obstet. Gynaecol..

[168] Appleyard C.B., Flores I., Torres-Reverón A. (2020). The Link Between Stress and Endometriosis: From Animal Models to the Clinical Scenario. Reprod. Sci..

[169] Orsolini L., Pompili S., Tempia Valenta S., Salvi V., Volpe U. (2022). C-Reactive Protein as a Biomarker for Major Depressive Disorder?. Int. J. Mol. Sci..

[170] Castrén E., Monteggia L.M. (2021). Brain-Derived Neurotrophic Factor Signaling in Depression and Antidepressant Action. Biol. Psychiatry.

[171] Sonmez E.O., Uguz F., Sahingoz M., Sonmez G., Kaya N., Camkurt M.A., Gokmen Z., Basaran M., Gezginc K., Erdem S.S. (2019). Effect of Maternal Depression on Brain-derived Neurotrophic Factor Levels in Fetal Cord Blood. Clin. Psychopharmacol. Neurosci..

[172] Rai S. (2025). Microbial Mysteries—Exploring the Microbiome\’s Impact on Endometriosis: A Review. J. South Asian Fed. Obstet. Gynaecol..

[173] Guo C., Zhang C. (2024). Role of the gut microbiota in the pathogenesis of endometriosis: A review. Front. Microbiol..

[174] Jiang I., Yong P.J., Allaire C., Bedaiwy M.A. (2021). Intricate Connections between the Microbiota and Endometriosis. Int. J. Mol. Sci..

[175] Zhou J.Z., Way S.S., Chen K. (2018). Immunology of the Uterine and Vaginal Mucosae. Trends Immunol..

[176] Weber I., Sienko A., Urban A., Szwed C., Czajkowski K., Basta P., Sienko J. (2024). Relationship between the gut microbiome and endometriosis and its role in pathogenesis, diagnosis, and treatment: A systematic review. Ginekol. Pol..

[177] Wang Y., Yan H., Zheng Q., Sun X. (2025). Crucial functions of gut microbiota on gut–liver repair. hLife.

[178] Di Vincenzo F., Del Gaudio A., Petito V., Lopetuso L.R., Scaldaferri F. (2024). Gut microbiota, intestinal permeability, and systemic inflammation: A narrative review. Intern. Emerg. Med..

[179] Neri B., Russo C., Mossa M., Martire F.G., Selntigia A., Mancone R., Calabrese E., Rizzo G., Exacoustos C., Biancone L. (2023). High Frequency of Deep Infiltrating Endometriosis in Patients with Inflammatory Bowel Disease: A Nested Case-Control Study. Dig. Dis..

[180] Gan R., Yi Y., Li Y. (2023). Association of endometriosis and inflammatory bowel disease (ibd), findings from epidemiological evidence to genetic links. Fertil. Steril..

[181] Rondanelli M., Borromeo S., Cavioni A., Gasparri C., Gattone I., Genovese E., Lazzarotti A., Minonne L., Moroni A., Patelli Z. (2025). Therapeutic Strategies to Modulate Gut Microbial Health: Approaches for Chronic Metabolic Disorder Management. Metabolites.

[182] Escorcia Mora P., Valbuena D., Diez-Juan A. (2025). The Role of the Gut Microbiota in Female Reproductive and Gynecological Health: Insights into Endometrial Signaling Pathways. Life.

[183] Hu S., Ding Q., Zhang W., Kang M., Ma J., Zhao L. (2023). Gut microbial beta-glucuronidase: A vital regulator in female estrogen metabolism. Gut Microbes.

[184] Wei Y., Tan H., Yang R., Yang F., Liu D., Huang B., OuYang L., Lei S., Wang Z., Jiang S. (2023). Gut dysbiosis-derived β-glucuronidase promotes the development of endometriosis. Fertil. Steril..

[185] Hases L., Stepanauskaite L., Birgersson M., Brusselaers N., Schuppe-Koistinen I., Archer A., Engstrand L., Williams C. (2023). High-fat diet and estrogen modulate the gut microbiota in a sex-dependent manner in mice. Commun. Biol..

[186] Caretto M., Simoncini T. (2021). Progestin or anti-estrogen treatment for endometrial cancer: Choosing the best option for selected patients. Gynecol. Endocrinol..

[187] Huang L., Liu B., Liu Z., Feng W., Liu M., Wang Y., Peng D., Fu X., Zhu H., Cui Z. (2021). Gut Microbiota Exceeds Cervical Microbiota for Early Diagnosis of Endometriosis. Front. Cell Infect. Microbiol..

[188] Chadchan S.B., Naik S.K., Popli P., Talwar C., Putluri S., Ambati C.R., Lint M.A., Kau A.L., Stallings C.L., Kommagani R. (2023). Gut microbiota and microbiota-derived metabolites promotes endometriosis. Cell Death Discov..

[189] Chompre G., Cruz M.L., Arroyo G.A., Rivera R.M., Colon M.C., Appleyard C.B. (2018). Probiotic Administration in an Endometriosis Animal Model Can Influence the Gut Microbiota and Gut-Brain Axis to Counteract the Effects of Stress. FASEB J..

[190] Zhang B., Mohd Sahardi N.F.N., Di W., Long X., Shafiee M.N. (2025). The Gut–Endometrium Axis: Exploring the Role of Microbiome in the Pathogenesis and Treatment of Endometrial Cancer—A Narrative Review. Cancers.

[191] Chen P., Guo Y., Jia L., Wan J., He T., Fang C., Li T. (2021). Interaction Between Functionally Activate Endometrial Microbiota and Host Gene Regulation in Endometrial Cancer. Front. Cell Dev. Biol..

[192] Wang R., Yang X., Liu J., Zhong F., Zhang C., Chen Y., Sun T., Ji C., Ma D. (2022). Gut microbiota regulates acute myeloid leukaemia via alteration of intestinal barrier function mediated by butyrate. Nat. Commun..

[193] Sinha S.R., Haileselassie Y., Nguyen L.P., Tropini C., Wang M., Becker L.S., Sim D., Jarr K., Spear E.T., Singh G. (2020). Dysbiosis-Induced Secondary Bile Acid Deficiency Promotes Intestinal Inflammation. Cell Host Microbe.

[194] Ma W., Mao Q., Xia W., Dong G., Yu C., Jiang F. (2019). Gut Microbiota Shapes the Efficiency of Cancer Therapy. Front. Microbiol..

[195] Alli S.R., Gorbovskaya I., Liu J.C.W., Kolla N.J., Brown L., Müller D.J. (2022). The Gut Microbiome in Depression and Potential Benefit of Prebiotics, Probiotics and Synbiotics: A Systematic Review of Clinical Trials and Observational Studies. Int. J. Mol. Sci..

[196] Sui Y., Wu J., Chen J. (2021). The Role of Gut Microbial β-Glucuronidase in Estrogen Reactivation and Breast Cancer. Front. Cell Dev. Biol..

[197] Pérez-Prieto I., Vargas E., Salas-Espejo E., Lüll K., Canha-Gouveia A., Pérez L.A., Fontes J., Salumets A., Andreson R., Aasmets O. (2024). Gut microbiome in endometriosis: A cohort study on 1000 individuals. BMC Med..

[198] Moustakli E., Zagorianakou N., Makrydimas S., Oikonomou E.D., Miltiadous A., Makrydimas G. (2025). The Gut–Endometriosis Axis: Genetic Mechanisms and Public Health Implications. Genes.

[199] Liang Z., Wu Q., Wang H., Tan J., Wang H., Gou Y., Cao Y., Li Z., Zhang Z. (2022). Silencing of lncRNA MALAT1 facilitates erastin-induced ferroptosis in endometriosis through miR-145-5p/MUC1 signaling. Cell Death Discov..

[200] Hudson Q.J., Proestling K., Perricos A., Kuessel L., Husslein H., Wenzl R., Yotova I. (2021). The Role of Long Non-Coding RNAs in Endometriosis. Int. J. Mol. Sci..

[201] Zhang L., Yu Z., Qu Q., Li X., Lu X., Zhang H. (2022). Exosomal lncRNA HOTAIR Promotes the Progression and Angiogenesis of Endometriosis via the miR-761/HDAC1 Axis and Activation of STAT3-Mediated Inflammation. Int. J. Nanomed..

[202] Ghazal S., McKinnon B., Zhou J., Mueller M., Men Y., Yang L., Mueller M., Flannery C., Huang Y., Taylor H.S. (2015). H19 lncRNA alters stromal cell growth via IGF signaling in the endometrium of women with endometriosis. EMBO Mol. Med..

[203] Li Z., Wei D., Yang C., Sun H., Lu T., Kuang D. (2016). Overexpression of long noncoding RNA, NEAT1 promotes cell proliferation, invasion and migration in endometrial endometrioid adenocarcinoma. Biomed. Pharmacother..

[204] Shan S., Yang Y., Jiang J., Yang B., Yang Y., Sun F., Zhang J., Lin Y., Xu H. (2022). Extracellular vesicle-derived long non-coding RNA as circulating biomarkers for endometriosis. Reprod. Biomed. Online.

[205] Frisendahl C., Tang Y., Boggavarapu N.R., Peters M., Lalitkumar P.G., Piltonen T.T., Arffman R.K., Salumets A., Götte M., Korsching E. (2024). miR-193b-5p and miR-374b-5p Are Aberrantly Expressed in Endometriosis and Suppress Endometrial Cell Migration In Vitro. Biomolecules.

[206] Hawkins S.M., Creighton C.J., Han D.Y., Zariff A., Anderson M.L., Gunaratne P.H., Matzuk M.M. (2011). Functional MicroRNA Involved in Endometriosis. Mol. Endocrinol..

[207] Di Pietro C., Caruso S., Battaglia R., Iraci Sareri M., La Ferlita A., Strino F., Bonaventura G., Di Mauro M., Barcellona M.L., Perciavalle V. (2018). MiR-27a-3p and miR-124-3p, upregulated in endometrium and serum from women affected by Chronic Endometritis, are new potential molecular markers of endometrial receptivity. Am. J. Reprod. Immunol..

[208] Canlorbe G., Wang Z., Laas E., Bendifallah S., Castela M., Lefevre M., Chabbert-Buffet N., Daraï E., Aractingi S., Méhats C. (2016). Identification of microRNA expression profile related to lymph node status in women with early-stage grade 1–2 endometrial cancer. Mod. Pathol..

[209] Ravaggi A., Bergamaschi C., Galbiati C., Zanotti L., Fabricio A.S.C., Gion M., Cappelletto E., Leon A.E., Gennarelli M., Romagnolo C. (2024). Circulating Serum Micro-RNA as Non-Invasive Diagnostic Biomarkers of Endometriosis. Biomedicines.

[210] Sasamoto N., Ngo L., Vitonis A.F., Dillon S.T., Missmer S.A., Libermann T.A., Terry K.L. (2022). Circulating proteomic profiles associated with endometriosis in adolescents and young adults. Hum. Reprod..

[211] Anchan M.M., Dutta R. (2025). Reframing Endometriosis: Interplay of NETs, Macrophages, and Lymphocytes at the Crossroads of Disease Progression, Infertility, and Malignant Transformation. Am. J. Reprod. Immunol..

[212] Krygere L., Jukna P., Jariene K., Drejeriene E. (2024). Diagnostic Potential of Cytokine Biomarkers in Endometriosis: Challenges and Insights. Biomedicines.

[213] Janša V., Klančič T., Pušić M., Klein M., Vrtačnik Bokal E., Ban Frangež H., Rižner T.L. (2021). Proteomic analysis of peritoneal fluid identified COMP and TGFBI as new candidate biomarkers for endometriosis. Sci. Rep..

[214] Ashish A., Mishra S., Rai S., Kusum K., Rai G., Singh R. (2024). Advances in Endometriosis Research: From Pathogenesis to Prevention. A Comprehensive Overview of Endometriosis.

[215] Erraji H., El Ghanmi A., Louanjli N., Benahmed M., El Mansouri F., Zarqaoui M., Ghazi B. (2025). Leveraging epigenetic aberrations in the pathogenesis of endometriosis: From DNA methylation to non-coding RNAs. Front. Genet..

[216] Choi M.R., Chang H.J., Heo J.-H., Yum S.H., Jo E., Kim M., Lee S.-R. (2024). Expression Profiling of Coding and Noncoding RNAs in the Endometrium of Patients with Endometriosis. Int. J. Mol. Sci..

[217] Azeze G.G., Wu L., Alemu B.K., Lee W.F., Fung L.W.Y., Cheung E.C.W., Zhang T., Wang C.C. (2024). Proteomics approach to discovering non-invasive diagnostic biomarkers and understanding the pathogenesis of endometriosis: A systematic review and meta-analysis. J. Transl. Med..

[218] Ortiz C.N., Torres-Reverón A., Appleyard C.B. (2021). Metabolomics in endometriosis: Challenges and perspectives for future studies. Reprod. Fertil..

[219] Ou Y., Wang H., Zhou C., Chen Y., Lyu J., Feng M., Huang X. (2025). Endometriosis-associated infertility: Multi-omics insights into pathogenesis and precision therapeutics. Front. Endocrinol..

[220] Bae S.-J., Jo Y., Cho M.K., Jin J.-S., Kim J.-Y., Shim J., Kim Y.H., Park J.-K., Ryu D., Lee H.J. (2022). Identification and analysis of novel endometriosis biomarkers via integrative bioinformatics. Front. Endocrinol..

[221] Sivajohan B., Elgendi M., Menon C., Allaire C., Yong P., Bedaiwy M.A. (2022). Clinical use of artificial intelligence in endometriosis: A scoping review. NPJ Digit. Med..

[222] Da Silva A.S., Anwar S., Park S., Park S., Goodfellow L., Sergaki C. (2025). The untapped potential of vaginal microbiome diagnostics for improving women’s health. Front. Cell Infect. Microbiol..

[223] Hajjo R., Sabbah D.A., Al Bawab A.Q. (2022). Unlocking the Potential of the Human Microbiome for Identifying Disease Diagnostic Biomarkers. Diagnostics.

[224] Hudson Q.J., Perricos A., Wenzl R., Yotova I. (2020). Challenges in uncovering non-invasive biomarkers of endometriosis. Exp. Biol. Med..

[225] Ronsini C., Fumiento P., Iavarone I., Greco P.F., Cobellis L., De Franciscis P. (2023). Liquid Biopsy in Endometriosis: A Systematic Review. Int. J. Mol. Sci..

[226] Zhang Y., Tian L. (2024). Advances and challenges in the use of liquid biopsy in gynaecological oncology. Heliyon.

[227] Barra F., Scala C., Ferrero S. (2018). Current understanding on pharmacokinetics, clinical efficacy and safety of progestins for treating pain associated to endometriosis. Expert Opin. Drug Metab. Toxicol..

[228] Reis F.M., Coutinho L.M., Vannuccini S., Batteux F., Chapron C., Petraglia F. (2020). Progesterone receptor ligands for the treatment of endometriosis: The mechanisms behind therapeutic success and failure. Hum. Reprod. Update.

[229] Flores V.A., Vanhie A., Dang T., Taylor H.S. (2018). Progesterone Receptor Status Predicts Response to Progestin Therapy in Endometriosis. J. Clin. Endocrinol. Metab..

[230] Ebert A.D., Dong L., Merz M., Kirsch B., Francuski M., Böttcher B., Roman H., Suvitie P., Hlavackova O., Gude K. (2017). Dienogest 2 mg Daily in the Treatment of Adolescents with Clinically Suspected Endometriosis: The VISanne Study to Assess Safety in ADOlescents. J. Pediatr. Adolesc. Gynecol..

[231] Römer T. (2018). Long-term treatment of endometriosis with dienogest: Retrospective analysis of efficacy and safety in clinical practice. Arch. Gynecol. Obstet..

[232] Johnson N.P., Hummelshoj L., Adamson G.D., Keckstein J., Taylor H.S., Abrao M.S., Bush D., Kiesel L., Tamimi R., Sharpe-Timms K.L. (2017). World Endometriosis Society consensus on the classification of endometriosis. Hum. Reprod..

[233] Piriyev E., Schiermeier S., Römer T. (2025). Hormonal Treatment of Endometriosis: A Narrative Review. Pharmaceuticals.

[234] Langlade C., Gouverneur A., Bosco-Lévy P., Gouraud A., Pérault-Pochat M., Béné J., Miremont-Salamé G., Pariente A. (2019). French Network of Pharmacovigilance Centres. Adverse events reported for Mirena levonorgestrel-releasing intrauterine device in France and impact of media coverage. Br. J. Clin. Pharmacol..

[235] Tosti C., Biscione A., Morgante G., Bifulco G., Luisi S., Petraglia F. (2017). Hormonal therapy for endometriosis: From molecular research to bedside. Eur. J. Obstet. Gynecol. Reprod. Biol..

[236] Giudice L.C., As-Sanie S., Arjona Ferreira J.C., Becker C.M., Abrao M.S., Lessey B.A., Brown E., Dynowski K., Wilk K., Li Y. (2022). Once daily oral relugolix combination therapy versus placebo in patients with endometriosis-associated pain: Two replicate phase 3, randomised, double-blind, studies (SPIRIT 1 and 2). Lancet.

[237] Donnez J., Becker C., Taylor H., Carmona Herrera F., Donnez O., Horne A., Paszkowski M., Petraglia F., Renner S.P., Patel A. (2024). Linzagolix therapy versus a placebo in patients with endometriosis-associated pain: A prospective, randomized, double-blind, Phase 3 study (EDELWEISS 3). Hum. Reprod..

[238] Becker C.M., Johnson N.P., As-Sanie S., Arjona Ferreira J.C., Abrao M.S., Wilk K., Imm S.J., Mathur V., Perry J.S., Wagman R.B. (2024). Two-year efficacy and safety of relugolix combination therapy in women with endometriosis-associated pain: SPIRIT open-label extension study. Hum. Reprod..

[239] Mitchell J.-B., Chetty S., Kathrada F. (2022). Progestins in the symptomatic management of endometriosis: A meta-analysis on their effectiveness and safety. BMC Womens Health.

[240] Barra F., Laganà A.S., Casarin J., Ghezzi F., Ferro Desideri L., Scala C., Ferrero S. (2019). Molecular Targets for Endometriosis Therapy: Where We Are and Where We Are Going?. Int. J. Fertil. Steril..

[241] Hull M.L., Charnock-Jones D.S., Chan C.L.K., Bruner-Tran K.L., Osteen K.G., Tom B.D.M., Fan T.-P.D., Smith S.K. (2003). Antiangiogenic Agents Are Effective Inhibitors of Endometriosis. J. Clin. Endocrinol. Metab..

[242] Nap A.W., Griffioen A.W., Dunselman G.A.J., Bouma-Ter Steege J.C.A., Thijssen V.L.J.L., Evers J.L.H., Groothuis P.G. (2004). Antiangiogenesis Therapy for Endometriosis. J. Clin. Endocrinol. Metab..

[243] Kapoor R., Stratopoulou C.A., Dolmans M.-M. (2021). Pathogenesis of Endometriosis: New Insights into Prospective Therapies. Int. J. Mol. Sci..

[244] Tejada M.Á., Santos-Llamas A.I., Fernández-Ramírez M.J., Tarín J.J., Cano A., Gómez R. (2021). A Reassessment of the Therapeutic Potential of a Dopamine Receptor 2 Agonist (D2-AG) in Endometriosis by Comparison against a Standardized Antiangiogenic Treatment. Biomedicines.

[245] Pellicer N., Galliano D., Herraiz S., Bagger Y.Z., Arce J.-C., Pellicer A. (2021). Use of dopamine agonists to target angiogenesis in women with endometriosis. Hum. Reprod..

[246] Cakmak B., Cavusoglu T., Ates U., Meral A., Nacar M.C., Erbaş O. (2015). Regression of experimental endometriotic implants in a rat model with the angiotensin II receptor blocker losartan. J. Obstet. Gynaecol. Res..

[247] Ramos-Nino M.E. (2025). Non-Hormonal Strategies in Endometriosis: Targets with Future Clinical Potential. J. Clin. Med..

[248] Dolmans M.-M., Donnez J. (2022). Emerging Drug Targets for Endometriosis. Biomolecules.

[249] Wang L., Tan Y.J., Wang M., Chen Y.F., Li X.Y. (2019). DNA Methylation Inhibitor 5-Aza-2′-Deoxycytidine Modulates Endometrial Receptivity Through Upregulating HOXA10 Expression. Reprod. Sci..

[250] Hsiao K., Wu M., Tsai S. (2017). Epigenetic regulation of the pathological process in endometriosis. Reprod. Med. Biol..

[251] Cacciottola L., Donnez J., Dolmans M.-M. (2021). Can Endometriosis-Related Oxidative Stress Pave the Way for New Treatment Targets?. Int. J. Mol. Sci..

[252] Konrad L., Dietze R., Riaz M.A., Scheiner-Bobis G., Behnke J., Horné F., Hoerscher A., Reising C., Meinhold-Heerlein I. (2020). Epithelial–Mesenchymal Transition in Endometriosis—When Does It Happen?. J. Clin. Med..

[253] Liu H., Zhu Z., Chen X., Lu J., Song Y., Xia W. (2023). A review of the effects of estrogen and epithelial-mesenchymal transformation on intrauterine adhesion and endometriosis. Transpl. Immunol..

[254] Lin Y.-K., Li Y.-Y., Li Y., Li D.-J., Wang X.-L., Wang L., Yu M., Zhu Y.-Z., Cheng J.-J., Du M.-R. (2022). SCM-198 Prevents Endometriosis by Reversing Low Autophagy of Endometrial Stromal Cell via Balancing ERα and PR Signals. Front. Endocrinol..

[255] Barra F., Scala C., Mais V., Guerriero S., Ferrero S. (2018). Investigational drugs for the treatment of endometriosis, an update on recent developments. Expert. Opin. Investig. Drugs.

[256] Ścieżyńska A., Komorowski M., Soszyńska M., Malejczyk J. (2019). NK Cells as Potential Targets for Immunotherapy in Endometriosis. J. Clin. Med..

[257] Artemova D., Vishnyakova P., Khashchenko E., Elchaninov A., Sukhikh G., Fatkhudinov T. (2021). Endometriosis and Cancer: Exploring the Role of Macrophages. Int. J. Mol. Sci..

[258] Zhang W., Li K., Jian A., Zhang G., Zhang X. (2024). Prospects for potential therapy targeting immune-associated factors in endometriosis (Review). Mol. Med. Rep..

[259] Chen Z., Yang Y., Liu L.L., Lundqvist A. (2019). Strategies to Augment Natural Killer (NK) Cell Activity against Solid Tumors. Cancers.

[260] Memon H., Patel B.M. (2019). Immune checkpoint inhibitors in non-small cell lung cancer: A bird’s eye view. Life Sci..

[261] Giannopoulos K. (2019). Targeting Immune Signaling Checkpoints in Acute Myeloid Leukemia. J. Clin. Med..

[262] Regis S., Dondero A., Caliendo F., Bottino C., Castriconi R. (2020). NK Cell Function Regulation by TGF-β-Induced Epigenetic Mechanisms. Front. Immunol..

[263] Yoshimura A., Wakabayashi Y., Mori T. (2010). Cellular and molecular basis for the regulation of inflammation by TGF-β. J. Biochem..

[264] Chang C.Y.-Y., Chiang A.-J., Yan M.-J., Lai M.-T., Su Y.-Y., Huang H.-Y., Chang C.-Y., Li Y.-H., Li P.-F., Chen C.-M. (2022). Ribosome Biogenesis Serves as a Therapeutic Target for Treating Endometriosis and the Associated Complications. Biomedicines.

[265] Kolahdouz-Mohammadi R., Shidfar F., Khodaverdi S., Arablou T., Heidari S., Rashidi N., Delbandi A. (2021). Resveratrol treatment reduces expression of MCP-1, IL-6, IL-8 and RANTES in endometriotic stromal cells. J. Cell Mol. Med..

[266] Vallée A., Lecarpentier Y. (2020). Curcumin and Endometriosis. Int. J. Mol. Sci..

[267] Takaoka O., Mori T., Ito F., Okimura H., Kataoka H., Tanaka Y., Koshiba A., Kusuki I., Shigehiro S., Amami T. (2018). Daidzein-rich isoflavone aglycones inhibit cell growth and inflammation in endometriosis. J. Steroid Biochem. Mol. Biol..

[268] Wei X., Shao X. (2018). Nobiletin alleviates endometriosis via down-regulating NF-κB activity in endometriosis mouse model. Biosci. Rep..

[269] Huang R., Chen S., Zhao M., Li Z., Zhu L. (2020). Ginsenoside Rg3 attenuates endometriosis by inhibiting the viability of human ectopic endometrial stromal cells through the nuclear factor-kappaB signaling pathway. J. Gynecol. Obstet. Hum. Reprod..

[270] Horie S., Harada T., Mitsunari M., Taniguchi F., Iwabe T., Terakawa N. (2005). Progesterone and progestational compounds attenuate tumor necrosis factor alpha–induced interleukin-8 production via nuclear factor kappaB inactivation in endometriotic stromal cells. Fertil. Steril..

[271] Yagyu T., Kobayashi H., Matsuzaki H., Wakahara K., Kondo T., Kurita N., Sekino H., Inagaki K., Suzuki M., Kanayama N. (2005). Thalidomide Inhibits Tumor Necrosis Factor-α-Induced Interleukin-8 Expression in Endometriotic Stromal Cells, Possibly through Suppression of Nuclear Factor-κB Activation. J. Clin. Endocrinol. Metab..

[272] El-Zayadi A.A., Mohamed S.A., Arafa M., Mohammed S.M., Zayed A., Abdelhafez M.S., Badawy A.M. (2020). Anti-IL-6 receptor monoclonal antibody as a new treatment of endometriosis. Immunol. Res..

[273] Gómez R., Abad A., Delgado F., Tamarit S., Simón C., Pellicer A. (2011). Effects of hyperprolactinemia treatment with the dopamine agonist quinagolide on endometriotic lesions in patients with endometriosis-associated hyperprolactinemia. Fertil. Steril..

[274] Chang L.-C., Chiang Y.-F., Chen H.-Y., Huang Y.-J., Liu A.-C., Hsia S.-M. (2020). The Potential Effect of Fucoidan on Inhibiting Epithelial-to-Mesenchymal Transition, Proliferation, and Increase in Apoptosis for Endometriosis Treatment: In Vivo and In Vitro Study. Biomedicines.

[275] Karamian A., Paktinat S., Esfandyari S., Nazarian H., Ziai S.A., Zarnani A.-H., Salehpour S., Hosseinirad H., Karamian A., Novin M.G. (2021). Pyrvinium pamoate induces in-vitro suppression of IL-6 and IL-8 produced by human endometriotic stromal cells. Hum. Exp. Toxicol..

[276] Hsu Y.-W., Chen H.-Y., Chiang Y.-F., Chang L.-C., Lin P.-H., Hsia S.-M. (2020). The effects of isoliquiritigenin on endometriosis in vivo and in vitro study. Phytomedicine.

[277] Yu M.-M., Zhou Q.-M. (2018). 3,6-dihydroxyflavone suppresses the epithelial-mesenchymal transition, migration and invasion in endometrial stromal cells by inhibiting the Notch signaling pathway. Eur. Rev. Med. Pharmacol. Sci..

[278] Qi S., Yan L., Liu Z., Mu Y., Li M., Zhao X., Chen Z.-J., Zhang H. (2018). Melatonin inhibits 17β-estradiol-induced migration, invasion and epithelial-mesenchymal transition in normal and endometriotic endometrial epithelial cells. Reprod. Biol. Endocrinol..

[279] Brichant G., Laraki I., Henry L., Munaut C., Nisolle M. (2021). New Therapeutics in Endometriosis: A Review of Hormonal, Non-Hormonal, and Non-Coding RNA Treatments. Int. J. Mol. Sci..

[280] Slayden O., Luo F., Park Y., Moses A.S., Demessie A.A., Singh P., Korzun T., Taratula O., Taratula O. (2024). Targeted nanoparticles for imaging and therapy of endometriosis. Biol. Reprod..

[281] Zhou C., Feng M., Chen Y., Lv S., Zhang Y., Chen J., Zhang R., Huang X. (2023). Unraveling immunotherapeutic targets for endometriosis: A transcriptomic and single-cell analysis. Front. Immunol..

[282] Koss M., Ziomek W., Bebrysz E., Palmi J., Dębek-Kalinowska K., Bartnik P., Baran J., Dunder I., Biszewski M., Drabik A. (2025). ADVANCES IN ENDOMETRIOSIS THERAPY: A REVIEW OF TARGETED THERAPIES, NANOPARTICLES AND STEM CELLS. Int. J. Innov. Technol. Soc. Sci..

[283] Zhu S., Zhang J., Xue N., Zhu X., Li F., Dai Q., Qing X., Chen D., Liu X., Wei Z. (2023). Highly specific neutrophil-mediated delivery of albumin nanoparticles to ectopic lesion for endometriosis therapy. J. Nanobiotechnol..

[284] Kiisholts K., Kurrikoff K., Arukuusk P., Porosk L., Peters M., Salumets A., Langel Ü. (2021). Cell-Penetrating Peptide and siRNA-Mediated Therapeutic Effects on Endometriosis and Cancer In Vitro Models. Pharmaceutics.

[285] Wu Y., Sun J., Li A., Chen D. (2018). The promoted delivery of RRM2 siRNA to vascular smooth muscle cells through liposome-polycation-DNA complex conjugated with cell penetrating peptides. Biomed. Pharmacother..

[286] Varela-Pombo C., López-Viñas B., Tustain S.Q., Maquieira C.G., Mateos J., Fafián-Labora J., Arufe M. (2025). Therapeutic application of mesenchymal stem cells in endometriosis. Life Sci..

[287] Cevik E.C., Mamillapalli R., Taylor H.S. (2025). Stem cells and female reproduction: Endometrial physiology, disease and therapy. Stem Cells.

[288] Chatzianagnosti S., Dermitzakis I., Theotokis P., Kousta E., Mastorakos G., Manthou M.E. (2024). Application of Mesenchymal Stem Cells in Female Infertility Treatment: Protocols and Preliminary Results. Life.

[289] Kong Y., Shao Y., Ren C., Yang G. (2021). Endometrial stem/progenitor cells and their roles in immunity, clinical application, and endometriosis. Stem Cell Res. Ther..

[290] Atiya H.I., Frisbie L., Goldfeld E., Orellana T., Donnellan N., Modugno F., Calderon M., Watkins S.C., Zhang R., Elishaev E. (2022). Endometriosis-Associated Mesenchymal Stem Cells Support Ovarian Clear Cell Carcinoma through Iron Regulation. Cancer Res..

[291] Liu J., Liu Q., Chen X. (2020). The Immunomodulatory Effects of Mesenchymal Stem Cells on Regulatory B Cells. Front. Immunol..

[292] Esfandyari S., Chugh R.M., Park H., Hobeika E., Ulin M., Al-Hendy A. (2020). Mesenchymal Stem Cells as a Bio Organ for Treatment of Female Infertility. Cells.

[293] de Miguel-Gómez L., López-Martínez S., Francés-Herrero E., Rodríguez-Eguren A., Pellicer A., Cervelló I. (2021). Stem Cells and the Endometrium: From the Discovery of Adult Stem Cells to Pre-Clinical Models. Cells.

[294] Bao C., Wang H., Fang H. (2022). Genomic Evidence Supports the Recognition of Endometriosis as an Inflammatory Systemic Disease and Reveals Disease-Specific Therapeutic Potentials of Targeting Neutrophil Degranulation. Front. Immunol..

[295] Zondervan K.T., Becker C.M., Koga K., Missmer S.A., Taylor R.N., Viganò P. (2018). Endometriosis. Nat. Rev. Dis. Primers.

[296] Chen L.-C., Hsu J.-W., Huang K.-L., Bai Y.-M., Su T.-P., Li C.-T., Yang A.C., Chang W.-H., Chen T.-J., Tsai S.-J. (2016). Risk of developing major depression and anxiety disorders among women with endometriosis: A longitudinal follow-up study. J. Affect. Disord..

[297] Alderman M., Yoder N., Taylor H. (2017). The Systemic Effects of Endometriosis. Semin. Reprod. Med..

[298] Viganò P., Ottolina J., Sarais V., Rebonato G., Somigliana E., Candiani M. (2018). Coagulation Status in Women With Endometriosis. Reprod. Sci..

[299] Tulandi T., Vercellini P. (2024). Growing evidence that endometriosis is a systemic disease. Reprod. Biomed. Online.

[300] Sanami S., Aghaamoo S., Ahmad S., Fazli A., Mansouri B., Nobre Oliveira J.I., Rahmanian M. (2025). Using systems biology and drug repositioning approaches to discover FDA-approved drugs candidates for endometriosis treatment. PLoS ONE.

[301] Starchenko A., Lauffenburger D.A. (2018). In vivo systems biology approaches to chronic immune/inflammatory pathophysiology. Curr. Opin. Biotechnol..

[302] Wu J., Fang X., Xia X. (2021). Identification of Key Genes and Pathways associated with Endometriosis by Weighted Gene Co-expression Network Analysis. Int. J. Med. Sci..

[303] Hickey S.L., McKim A., Mancuso C.A., Krishnan A. (2022). A network-based approach for isolating the chronic inflammation gene signatures underlying complex diseases towards finding new treatment opportunities. Front. Pharmacol..

[304] Zhang H., Mo Y., Wang L., Zhang H., Wu S., Sandai D., Shuid A.N., Chen X. (2024). Potential shared pathogenic mechanisms between endometriosis and inflammatory bowel disease indicate a strong initial effect of immune factors. Front. Immunol..

[305] Chen C.-C., Chou Y.-C., Hsu C.-Y., Tsai E.-M., Er T.-K. (2022). Transcriptome Profiling of Eutopic and Ectopic Endometrial Stromal Cells in Women with Endometriosis Based on High-Throughput Sequencing. Biomedicines.

[306] Wang Y., Chen Y., Xiao Y., Ruan J., Tian Q., Cheng Q., Chang K., Yi X. (2023). Distinct subtypes of endometriosis identified based on stromal-immune microenvironment and gene expression: Implications for hormone therapy. Front. Immunol..

[307] Sanches P.H.G., de Melo N.C., Porcari A.M., de Carvalho L.M. (2024). Integrating Molecular Perspectives: Strategies for Comprehensive Multi-Omics Integrative Data Analysis and Machine Learning Applications in Transcriptomics, Proteomics, and Metabolomics. Biology.

[308] Molla G., Bitew M. (2024). Revolutionizing Personalized Medicine: Synergy with Multi-Omics Data Generation, Main Hurdles, and Future Perspectives. Biomedicines.

[309] Hassan A.M., Naeem S.M., Eldosoky M.A.A., Mabrouk M.S. (2025). Multi-omics-based Machine Learning for the Subtype Classification of Breast Cancer. Arab. J. Sci. Eng..

[310] He S., Li H., Wan L., Qin X. (2025). Global landscape of clinical trials for endometriosis: Dynamic trends and future directions. Int. J. Surg..

[311] Zhan L., Cao Y. (2024). Personalized therapy in endometriosis—Based on ERα or ERβ expression. BMC Med..

[312] Becker C.M., Gattrell W.T., Gude K., Singh S.S. (2017). Reevaluating response and failure of medical treatment of endometriosis: A systematic review. Fertil. Steril..

[313] Cetera G.E., Merli C.E.M., Facchin F., Viganò P., Pesce E., Caprara F., Vercellini P. (2023). Non-response to first-line hormonal treatment for symptomatic endometriosis: Overcoming tunnel vision. A narrative review. BMC Womens Health.

[314] Herrara V., Tarab-Ravski D., Chauhan S.C., Narang N., Mirazul Islam M., Peer D., Prasad R., Yallapu M.M. (2025). Nanotechnology strategies for endometrium health: Are we on the right track?. Bioact. Mater..

[315] Luo X., Jia K., Xing J., Yi J. (2024). The utilization of nanotechnology in the female reproductive system and related disorders. Heliyon.

[316] Zhang P., Wang J., Miao J., Zhu P. (2025). The dual role of tissue regulatory T cells in tissue repair: Return to homeostasis or fibrosis. Front. Immunol..

[317] Merino J.J., Cabaña-Muñoz M.E. (2023). Nanoparticles and Mesenchymal Stem Cell (MSC) Therapy for Cancer Treatment: Focus on Nanocarriers and a si-RNA CXCR4 Chemokine Blocker as Strategies for Tumor Eradication In Vitro and In Vivo. Micromachines.

[318] Kłodnicka K., Michalska A., Januszewski J., Forma A., Teresiński G., Flieger J., Bogucki J., Maciejewski M., Syty K., Baj J. (2025). From Inflammation to Malignancy: The Link Between Endometriosis and Gynecological Cancers. Int. J. Mol. Sci..

[319] Chen L., Sererino E., Wu D.-S., Segars J., Shih I.-M. (2025). Somatic Cancer Driver Mutation Analysis in Endometriosis with Tumor-Like Presentations. Preprints.

[320] Karalis V.D. (2024). The Integration of Artificial Intelligence into Clinical Practice. Appl. Biosci..

[321] Wu C., Sun Y., Yang D., Peng H. (2025). Advances in endometrial receptivity and embryo implantation by multi-omics techniques. Anim. Zoonoses.

